# Secretome identification of immune cell factors mediating metastatic cell homing

**DOI:** 10.1038/srep17566

**Published:** 2015-12-04

**Authors:** Brian A. Aguado, Jia J. Wu, Samira M. Azarin, Dhaval Nanavati, Shreyas S. Rao, Grace G. Bushnell, Chaitanya B. Medicherla, Lonnie D. Shea

**Affiliations:** 1 Department of Biomedical Engineering, Northwestern University, Evanston, IL 60208, USA; 2 Simpson Querrey Institute for Bionanotechnology, Northwestern University, Chicago, IL 60611, USA; 3 Interdepartmental Biological Sciences, Northwestern University, Evanston, IL 60208, USA; 4 Department of Chemical Engineering and Materials Science, University of Minnesota, Minneapolis, MN 55455, USA; 5 Proteomics Core Facility, Northwestern University, Chicago, IL 60611, USA; 6 Department of Chemical and Biological Engineering, University of Alabama, Tuscaloosa, AL 35487, USA; 7 Department of Biomedical Engineering, University of Michigan, Ann Arbor, MI 48105, USA; 8 Feinberg School of Medicine, Northwestern University, Chicago, IL 60611, USA; 9 Department of Chemical Engineering, University of Michigan, Ann Arbor, MI 48105, USA; 10 Department of Chemical and Biological Engineering, Northwestern University, Evanston, IL 60208, USA

## Abstract

Metastatic cell homing is a complex process mediated in part by diffusible factors secreted from immune cells found at a pre-metastatic niche. We report on connecting secretomics and TRanscriptional Activity CEll aRray (TRACER) data to identify functional paracrine interactions between immune cells and metastatic cells as novel mediators of homing. Metastatic breast cancer mouse models were used to generate a diseased splenocyte conditioned media (D-SCM) containing immune cell secreted factors. MDA-MB-231 metastatic cell activity including cell invasion, migration, transendothelial migration, and proliferation were increased in D-SCM relative to control media. Our D-SCM secretome analysis yielded 144 secreted factor candidates that contribute to increased metastatic cell activity. The functional mediators of homing were identified using MetaCore software to determine interactions between the immune cell secretome and the TRACER-identified active transcription factors within metastatic cells. Among the 5 candidate homing factors identified, haptoglobin was selected and validated *in vitro* and *in vivo* as a key mediator of homing. Our studies demonstrate a novel systems biology approach to identify functional signaling factors associated with a cellular phenotype, which provides an enabling tool that complements large-scale protein identification provided by proteomics.

During cancer progression, the likelihood of patient survival significantly declines with the formation of metastatic tumors. Metastasis is a multi-step process, in which circulating tumor cells disseminate from the primary tumor and colonize distant organs. Prior to the formation of a metastatic lesion, a pre-metastatic niche is formed at a distant organ, which actively promotes metastatic cell homing to the site^1^. The formation of the niche begins when the primary tumor secretes factors and chemokines that mobilize inflammatory immune cells to the target organ^2^,^3^,^4^. Once recruited to the organ, immune cells subsequently secrete a multitude of factors both locally and distally, causing the homing of circulating tumor cells from the vasculature and lymphatic vessels^5^,^6^. The pre-metastatic niche increases the probability of tumor cell colonization and survival; therefore, strategies to effectively identify and target the factors that contribute to metastatic cell homing could be employed to limit tumor cell spreading to primed metastatic sites.

Crosstalk between immune cells at the niche and tumor cells has been implicated as a contributor for homing to the niche. Immune cells secrete a vast number of signaling molecules, and while a few chemokines have been described as contributors to homing^2^,^5^,^7^,^8^,^9^, techniques are needed to further identify functional secreted factors that promote homing. The need for efficiently identifying proteins that mediate a phenotypic response from a list of candidates is expanding due to the enabling capabilities provided by high-throughput strategies such as proteomics. In a specific application of proteomics termed secretomics, the initial protein list is filtered to screen for factors secreted via classical N-terminus signal recognition peptides or exosomal release^10^,^11^. Secretome analyses have identified several disease biomarkers, which are being developed as emerging therapies for breast cancer and other diseases^12^. Secretomics techniques typically catalog hundreds of candidate proteins; identifying the functional components that mediate changes in cell phenotype or disease state among the hundreds of candidates is traditionally accomplished through a combination of quantitative, abundance-based techniques^13^,^14^ and prediction-based computational approaches^15^. A methodology to more effectively narrow the pool of candidates and identify the proteins that mediate specific phenotypes, such as homing, could provide an enabling tool to address the expanding opportunities provided by proteomics.

In this report, we applied a novel systems biology strategy based on the computational intersection of secretomics and transcription factor (TF) activity to identify immune cell secreted factors that promote metastatic cell homing to the pre-metastatic niche. We stimulated MDA-MB-231 breast tumor cells using a splenocyte conditioned media (SCM) containing a complex mixture of immune cell secreted factors and induced phenotypic changes in metastatic cell activity. Using a secretomics approach, the immune cell secretome was analyzed to identify the secreted factors involved in activating the phenotypic changes in cancer cells. In parallel, we used a TRanscriptional Activity CEll aRray (TRACER) to identify active transcription factors (TFs) involved with the increased MDA-MB-231 metastatic activity in response to the secreted factors. Upon connecting the two data sets, the generated network connecting the SCM secreted factors to the activated TFs in TRACER was utilized to identify functional secreted factors that contribute to metastatic cell homing. One candidate secreted factor, haptoglobin, was validated *in vitro* and *in vivo* to confirm its role in metastatic cell homing. Connecting secretome and TRACER data provides a novel approach for identifying functional proteins within a secretome, which was validated through identifying proteins involved in metastatic cell homing.

## Results

### Immune cell secreted factors influence metastatic processes in MDA-MB-231 cells

**Figure 1:**
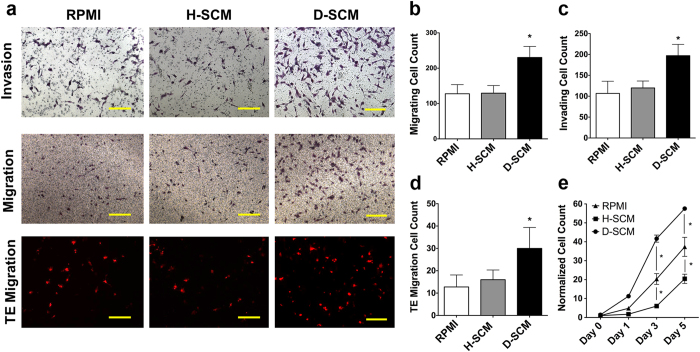
*In vitro* phenotypic changes of MDA-MB-231 cells in response to splenocyte conditioned media. (**a**) Representative bright field and fluorescence images of invading, migrating, and transendothelial (TE) migrating tumor cells in various media (scale bars = 300 μm). Tumor cell (**b**) migration, (**c**) invasion, and (**d**) TE migration cell counts per image in control RPMI, H-SCM, and D-SCM (*n* ≥ 8 for all assays, *p < 0.001 compared to RPMI and H-SCM). (**e**) MDA-MB-231 proliferation in various media measured using an MTS assay over five days (*n* = 12, *p < 0.001 compared to RPMI).

Metastatic cell processes under the influence of immune cell secreted factors were investigated using multiple *in vitro* phenotypic assays. Leukocytes were harvested from spleens of diseased mice (inoculated with breast cancer cells) and healthy mice (not inoculated with breast cancer cells), which are referred to as diseased and healthy spleens, respectively. Diseased spleens had an increased number of Gr1^hi^CD11b^+^ myeloid derived suppressor cells (MDSCs), decreased number of F4/80^+^ CD11b^+^ macrophages, and increased number of CD11c^+^ dendritic cells relative to healthy spleens (Supplementary Fig. 1). Given the differences in immune cell populations, splenocyte conditioned media from healthy (H-SCM) and diseased (D-SCM) splenocyte populations were generated. Differences in metastatic cell activity in H-SCM and D-SCM were evaluated using transwell culture assays. Representative images from the transwell assays are provided, which had increased MDA-MB-231 invasion, migration, and transendothelial migration when cultured in D-SCM compared to H-SCM and RPMI controls (Fig. 1A).

Increased migration and invasion of MDA-MB-231 cells was observed for culture with D-SCM relative to H-SCM or unconditioned RPMI media controls. Cell migration was quantified in D-SCM with 230.4 ± 11.1 cells per image compared to 127.8 ± 25.8 cells per image in RPMI. Cell migration was not significantly affected in H-SCM with 129.4 ± 21.5 cells per image (Fig. 1B). Similarly, MDA-MB-231 invasive cell counts in D-SCM were 197.0 ± 27.1 cells per image, which was significantly greater than the 119.9 ± 5.9 cells per image for H-SCM and 106.8 ± 29.2 cells per image in RPMI (Fig. 1C).

A human umbilical vein endothelial cell (HUVEC) monolayer was subsequently used to model extravasation of MDA-MB-231 cells in response to the conditioned media. MDA-MB-231 cells had increased migration through the endothelial barrier (30.0 ± 9.4 cells per image) in D-SCM compared to RPMI (12.8 ± 5.4 cells per image) and H-SCM (16.0 ± 4.3 cells per image) (Fig. 1D). Proliferation rates increased for cells grown in D-SCM, with significant increases in cell counts at Days 3 and 5 compared to cell numbers with culture in H-SCM at corresponding days (Fig. 1E). Taken together, D-SCM increased metastatic activity (i.e. cell processes associated with metastasis) in all tested assays compared to H-SCM and unconditioned media controls.

### Merging secretome and TRACER data yields candidate homing targets

**Figure 2:**
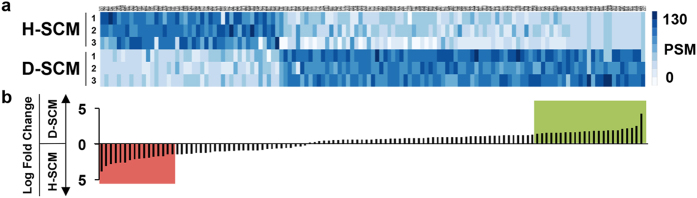
Secretome analysis of SCM. (**a**) Heat-map indicating peptide spectral matches (PSM) of 115 identified secreted factors for three replicates of H-SCM and D-SCM. (**b**) Log-fold change of corresponding secreted factor peptide hits. The green shaded region indicates secreted factors with a log-fold change greater than 1.5 in D-SCM and the red shaded region indicates secreted factors with a log-fold change greater than 1.5 in H-SCM. Log-fold differences and *p*-values for each comparison are provided in Supplementary Table 1.

Secretomics techniques were employed to identify the candidate immune cell secreted factors in D-SCM that increase metastatic activity of MDA-MB-231 cells *in vitro*. A total of 615 proteins were identified in both D-SCM and H-SCM, with 101 proteins identified exclusively in D-SCM and 139 proteins identified exclusively in H-SCM. Out of the 375 proteins identified in both media, 115 of those proteins were identified ontologically as secreted factors (Fig. 2A). From this secreted factor pool, 23 proteins in D-SCM and 16 proteins in H-SCM had a log2 fold change greater than 1.5, indicating increased protein abundance in the sample (Fig. 2B, Supplementary Table 1). In addition to the 115 secreted factors identified in both media, 29 of the 101 proteins exclusively present in D-SCM were identified as secreted factors and included in the secreted factor list, bringing the total to 144 secreted factors in D-SCM (Supplementary Table 1).

**Figure 3:**
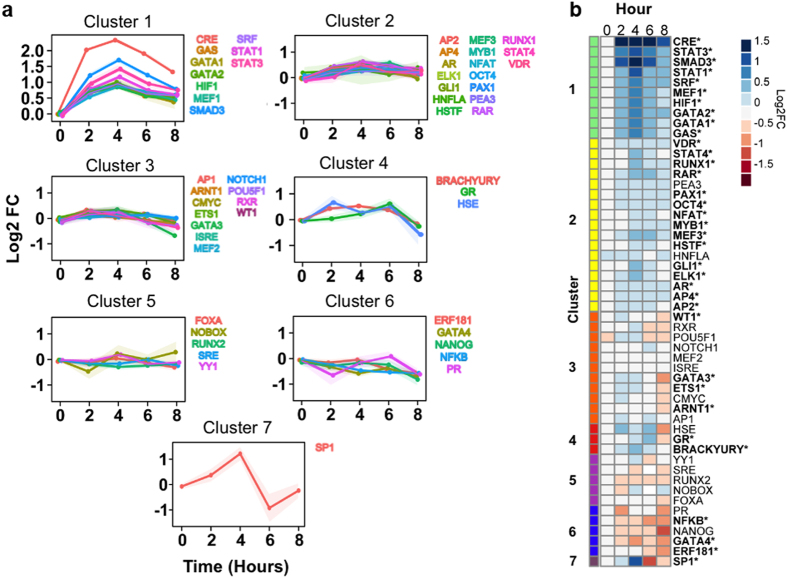
TRanscriptional Activity CEll aRray (TRACER) activity of MDA-MB-231 cells cultured in D-SCM. (**a**) *K*-cluster line-graphs for TF reporter activity of MDA-MB-231 cells cultured in D-SCM. (**b**) Heat map of normalized TF reporter activity values for MDA-MB-231 cells cultured in D-SCM over 8 hours. RPMI was chosen as a control for basal TF reporter activity since there was no observable difference in cell phenotype between MDA-MB-231 cells cultured in RPMI or H-SCM. Activity values are organized using *k*-means into 7 clusters (*n* = 6 arrays). Significance in TF activity for at least one time point indicated with an asterisk (*p < 0.05).

The transcription factors (TFs) activated in response to D-SCM were subsequently measured using a technique termed TRACER. The transactivation profiles of 52 TF reporter constructs over a time period of 8 hours were determined by measuring TF activity of cells cultured in D-SCM (Supplementary Fig. 2). Of the 52 TF reporters used in our TRACER analysis, 35 reporters had significantly altered TF activity (adjusted *p*-value < 0.05) for cells cultured in D-SCM. Using *k*-means clustering, TF activity profiles were grouped into 7 clusters, revealing TFs with similar temporal activation over the 8-hour period (Fig. 3A, Supplementary Fig. 3). The 7 clusters were organized from most to least active, allowing visualization of TF clusters that are most active in response to D-SCM secreted factors (Fig. 3B). The cluster with the greatest increase in reporter activation contained 10 TFs involved in metastatic cell processes including migration, proliferation, and invasion (Supplementary Table 2).

**Figure 4:**
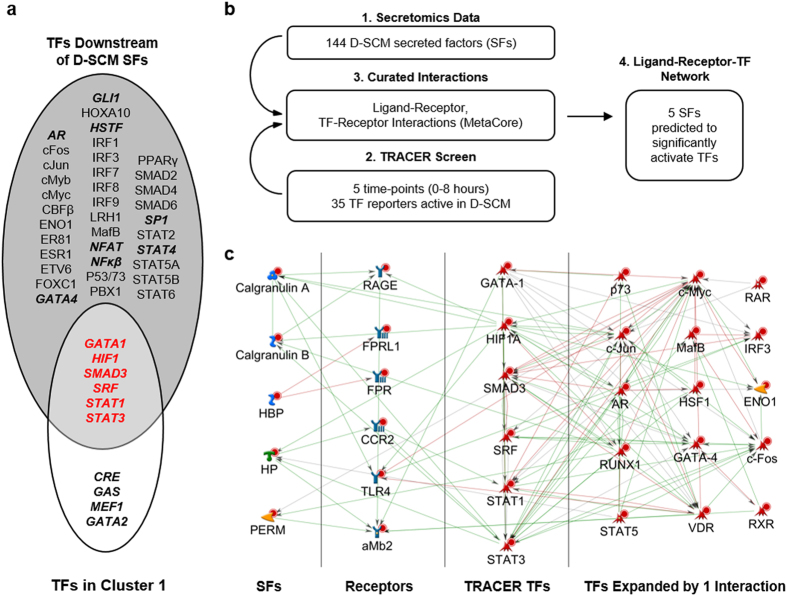
Identification of secreted factors and transcription factors mediating metastatic cell homing. (**a**) Venn diagram summarizing predicted TFs downstream of D-SCM. The white circle identifies the most active TFs (Cluster 1) in the D-SCM TRACER screen, and the grey circle identifies TFs that were predicted to be downstream of the secreted factors (SFs) identified using MetaCore. A Fisher test was used to test the significance of the overlapping TFs in red (*p* < 0.01). Bolded TFs indicate reporters that were significantly active in the D-SCM TRACER screen. (**b**) Overview of experimental approach and computational analysis to narrow down secreted factor list of candidates. (**c**) Network generated in MetaCore summarizing interactions between 5 secreted factor candidates, receptors, and downstream TFs from TRACER in Cluster 1. The network includes TFs expanded by one interaction.

Candidate homing factors were subsequently identified through the intersection of TFs downstream from D-SCM proteins and TFs identified with TRACER. First, 47 TFs were identified to be downstream of the 144 D-SCM secreted factors using public data sources that curate experimentally verified interactions. MetaCore network analysis software was used to generate a network containing 144 D-SCM secreted factors, all known human receptors, and all known human TFs as nodes. The group of 47 TFs predicted to be downstream of the secreted factors were compared to the 10 TFs within the cluster with the greatest increase in reporter activation identified with TRACER, revealing 6 common TF targets (Fig. 4A). We determined that the highly activated cluster of TFs was significantly enriched with TFs predicted to be downstream of 144 secreted factors.

Next, an interaction network was generated to determine functional connections between secreted factors and active TFs in TRACER. The 144 secreted factors, 35 significantly active TFs, and a list of all known human receptors obtained from MetaCore were connected as nodes to generate a network to link our secretomics and TRACER results (Fig. 4B). Edges between nodes represent experimentally verified protein-protein or gene-gene interactions. Additionally, the initial receptor and TF nodes were used as seed nodes and expanded by one degree to include additional signaling components. The final network consisted of 3562 known interactions. The 6 common TF targets were predicted to interact with receptors known to respond to 5 secreted factors identified in the network (calgranulin A, calgranulin B, haptoglobin, heme binding protein, and myeloperoxidase) (Fig. 4C, Supplementary Table 3). By narrowing the list of TFs included in the network with TRACER, we could objectively identify secreted factor candidates that have a downstream effect on the network.

### Haptoglobin identified as a secreted factor mediating tumor cell recruitment *in vitro*

**Figure 5:**
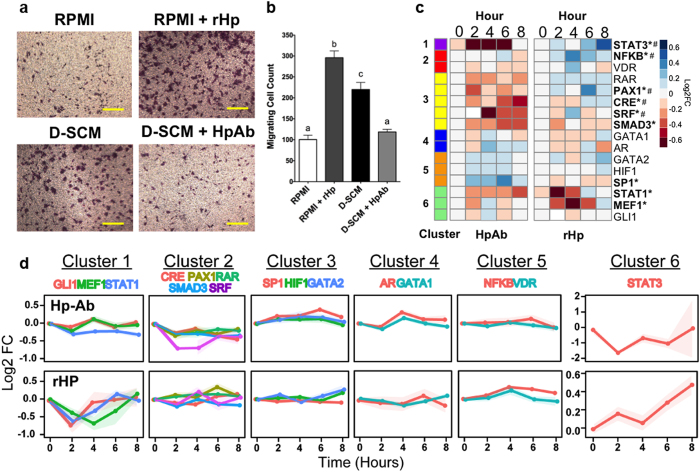
*In vitro* validation of haptoglobin as a secreted factor mediating MDA-MB-231 migration. (**a**) Representative migration assay images of MDA-MB-231 cells cultured in RPMI, RPMI supplemented with recombinant haptoglobin (RPMI + rHp), D-SCM, and D-SCM supplemented with haptoglobin antibody (D-SCM + HpAb) (scale bars = 300 μm). (**b**) Migrating cell count of MDA-MB-231 cells cultured with RPMI, RPMI + rHp, D-SCM, and D-SCM + HpAb (*n* = 8). Letters above each data column indicate statistical significance, with different letters signifying distinct statistical groups (*p* < 0.05). (**c**) TRACER array data showing MDA-MB-231 TF activity for cells cultured in D-SCM + HpAb normalized to activity for cells cultured in D-SCM, and activity for cells cultured in RPMI + rHp normalized to activity for cells cultured in RPMI (*n* = 6 arrays). Significant changes in TF activity for at least one time point indicated with an asterisk (*p < 0.05). TF reporters showing both increased activity in rHp and decreased activity in HpAb are indicated with a pound sign (#). (**d**) *K*-cluster line-graphs comparing TF activity clusters of MDA-MB-231 cells cultured in RPMI vs. rHp and D-SCM vs. D-SCM + HpAb.

Among the list of 5 candidate secreted factors, haptoglobin was chosen for validation. In the generated network, haptoglobin interacts with the CCR2 receptor and activates multiple downstream TFs (Supplementary Table 3). Western blotting confirmed the increased abundance of haptoglobin in D-SCM relative to H-SCM, with increased band density for three individual lots of D-SCM (Supplementary Fig. 4). Next, the role of haptoglobin on the *in vitro* metastatic activity of MDA-MB-231 cells was investigated using two approaches: i) RPMI supplemented with recombinant haptoglobin (rHp) relative to control RPMI media, and ii) D-SCM supplemented with a haptoglobin antibody (HpAb) relative to control D-SCM. MDA-MB-231 migration increased almost three-fold to 295.9 ± 16.9 cells in rHp supplemented medium compared to 100.5 ± 10.4 cells in control RPMI. Additionally, MDA-MB-231 migration was decreased almost two-fold in D-SCM supplemented with HpAb, with 118.6 ± 6.2 cells compared to 219.9 ± 16.9 cells in D-SCM. (Fig. 5A,B). MDA-MB-231 invasion also decreased in D-SCM supplemented with HpAb compared to D-SCM; however, invasion in RPMI supplemented with rHp was similar to control RPMI (Supplementary Fig. 5A). The addition of rHp to RPMI and HpAb to D-SCM had no effect on MDA-MB-231 transendothelial migration (Supplementary Fig. 5B). MDA-MB-231 cells cultured in rHp had a 1.5-fold increase in proliferation compared to RPMI by Day 5 of culture (Supplementary Fig. 5C). From these results, rHp showed differing effects on metastatic cell phenotype, suggesting other factors in the conditioned media contribute to the phenotypic effects.

Given these phenotypic changes in MDA-MB-231 metastatic processes, TRACER was employed to confirm the activity of TFs downstream of haptoglobin identified in the network. Two TRACER arrays were performed to compare TF activity between i) RPMI vs. RPMI + rHp (rHp TRACER) and ii) D-SCM vs. D-SCM + HpAb (HpAb TRACER). Reporters for the rHp and HpAb TRACER arrays were selected based on the TFs located downstream of the Hp-CCR2 interaction in the network. Reporters with increases in TF activity identified by TRACER for cells cultured in D-SCM were also selected. The transactivation profiles of 16 TF reporters were measured over a period of 8 hours (Supplementary Fig. 6). *K*-means clustering of the TRACER profiles characterized the response to rHp and HpAb treatments into 6 temporally distinct activity profiles (Fig. 5C,D, Supplementary Fig. 7). TF activity from 5 of 16 reporters (STAT3, NFκβ, PAX1, CRE, and SRF) correlated with recombinant haptoglobin addition, and displayed increased activity with rHp treatment while having decreased activity with HpAb treatment (Fig. 5C). Other reporters deviated from this trend, including SMAD3, SP1, STAT1, and MEF1, suggesting that other secreted factors in D-SCM may contribute to altering the activity of these TF reporters. These TRACER results demonstrated that haptoglobin activated multiple TFs associated with increased metastatic activity, and validated key TFs identified as downstream of Hp-CCR2 signaling from the network (Supplementary Table 3).

### Implanted biomaterial scaffolds releasing haptoglobin recruit tumor cells *in vivo*

**Figure 6:**
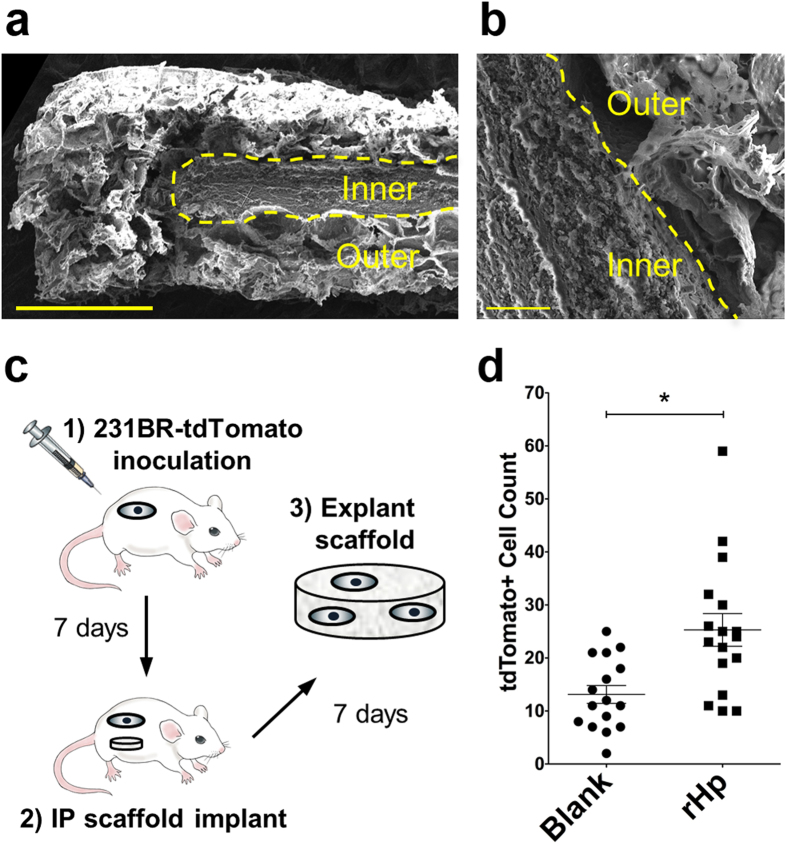
*In vivo* validation of haptoglobin as a secreted factor mediating MDA-MB-231BR cell homing. (**a**) SEM cross-section image of a haptoglobin layered scaffold (scale bar = 1 mm). (**b**) SEM image of inner and outer layer interface, indicated by a yellow dashed line (scale bar = 100 μm). (**c**) Schematic of *in vivo* study. NSG mice were inoculated with MDA-MB-231BR cells and implanted with a layered protein scaffold in the intraperitoneal (IP) fat 7 days later. The scaffold was explanted and cells were harvested for flow cytometry. Permission to use the syringe image^50^ was obtained from Nature Publishing Group. (**d**) Flow cytometry of scaffold cells indicating tumor cell counts on blank and rHp loaded scaffolds (*n* = 16, *p < 0.002).

The capability of haptoglobin to recruit tumor cells was tested in an orthotopic model of metastatic breast cancer^17^. A layered biomaterial scaffold composed of poly(lactide-co-glycolide) (PLG) was utilized to deliver haptoglobin locally from the implant. The scaffold consists of a packed inner layer of PLG microparticles containing lyophilized rHp, with a microporous outer layer. Scanning electron microscope images of the scaffold cross-section reveal the dense microstructure of the inner layer and the highly porous outer layer (Fig. 6A,B). The scaffolds were subsequently implanted in the intraperitoneal fat pad of NSG female mice, with a tdTomato labeled cell line derived from MDA-MB-231 cells delivered into the mammary fat pad. After one week, scaffolds were explanted and cells were harvested for flow cytometry analysis (Fig. 6C). PLG scaffolds without haptoglobin recruited 13.1 ± 1.7 tumor cells to the implant site. Upon adding rHp to the inner layer of the scaffold, tumor cell recruitment to the scaffold increased significantly to 25.3 ± 3.1 cells per scaffold (Fig. 6D). These results indicate that haptoglobin contributes to metastatic cell homing to the scaffold.

## Discussion

We describe a multi-disciplinary technique for identifying key mediators of paracrine signaling, which was applied to the interactions between immune cells and metastatic cancer cells. After identifying candidate mediators, our approach was validated by using the candidate factor to enhance metastatic cell homing to a biomaterial implant *in vivo*. Previous studies have identified secreted factors associated with promoting tumor cell homing to the pre-metastatic niche, including inflammatory cytokines^4^,^9^, ECM remodeling proteins^18^,^19^, and tumor-associated factors^7^. The identification of pathways contributing to metastasis has relied largely on utilizing gene expression profiling technologies^20^,^21^. Previous reports describe secretome analyses of primary breast cancer cells^22^, stromal cells^14^, and osteoblasts^23^ and identify large catalogs of proteins that may contribute to cancer cell homing and metastasis. However, transcriptomics and secretomics techniques measure relative abundances of RNA transcripts and secreted proteins respectively, and do not necessarily correlate to functional paracrine responses. We sought to apply a systems biology technique to connect relative abundances of secreted factors with downstream cellular activity and identify functional interactions due to paracrine signaling.

Our novel systems biology approach of connecting secretomics data with functional TF activity data from TRACER allows narrowing of candidate factors to identify functional secreted protein candidates involved in paracrine signaling. Immune cells reflective of the cell types that recruit tumor cells to the pre-metastatic niche were used to generate the D-SCM containing a mixture of functional secreted factors^17^,^24^,^25^. In pathological conditions such as metastasis, MDSC differentiation into mature granulocytes (i.e. macrophages and dendritic cells) is partially inhibited, thus allowing for an expansion of undifferentiated MDSCs and modulation of mature granulocyte populations at the spleen^26^. We observed phenotypic changes in MDA-MB-231 such as invasion, migration, transendothelial migration, and proliferation in response to the immune cell secreted factors. Secretomics analysis of the media identified 144 candidate factors that mediate the phenotypic changes, and this list was shortened through determining activated TFs within the cells. Using curated factor-receptor interactions to connect secretomics and the most active TFs identified by TRACER, we identified 5 secreted factors that are predicted to induce the observed TRACER activation profiles and MDA-MB-231 phenotype changes. TRACER has the capacity to evaluate TF activity over time in response to a variety of stimuli, including drugs^27^, 3D matrices^28^, and secreted proteins^16^. Our D-SCM TRACER screen identified a cluster of TFs that may be immediately downstream of secreted factors, given increases in activity on a short 8-hour time span. The 6 enriched TFs targets, including GATA1^29^, HIF1^30^, SMAD3^31^, SRF^32^, and STAT1/STAT3 proteins^33^, have been associated with metastasis, and herein, we demonstrate their role in mediating metastatic cell homing. TRACER allowed for a broad evaluation of paracrine signaling between immune cells and metastatic cells, and connecting together secretomics with TF activity data sets enabled the identification of functional secreted factors associated with metastatic cell homing.

Functional paracrine interactions between immune cells and metastatic cells identified using our strategy were validated experimentally *in vitro*. Our results identified haptoglobin as a potential mediator of metastatic cell recruitment. Haptoglobin is a secreted acute phase protein responsible for sequestering free hemoglobin in the circulation^34^,^35^, and has been implicated as a breast cancer disease marker^36^,^37^,^38^. While haptoglobin has been proposed as a diagnostic marker, these studies identify a mechanism by which it may contribute to metastatic spread. The increased presence of haptoglobin in the D-SCM is necessary for *in vitro* migration, given the significant decrease of migration when free haptoglobin in D-SCM was blocked with HpAb. The increased migration observed with rHp also supports the role of haptoglobin in MDA-MB-231 recruitment. In the generated network, the secreted factor haptoglobin interacts with CCR2^38^, which may cause a cascade of downstream TF activity associated with the increased migration of MDA-MB-231 cells^39^. These results highlight the role of extracellular haptoglobin on tumor cell homing, and contribute to increasing our understanding of homing mechanisms.

The role of haptoglobin in recruiting tumor cells to a defined niche was validated using an animal model of human metastatic breast cancer. We have previously reported on the ability of implanted PLG porous scaffolds to recruit circulating tumor cells *in vivo*, in which immune cells present at the scaffold modulated the implant microenvironment to prime the scaffold for tumor cell arrival^17^. This work further demonstrates that immune cells are capable of recruiting metastatic cells to a defined scaffold niche. These studies were performed with human triple negative breast cancer cells in immunocompromised mice, which lack cells from the adaptive immune system and allows us to focus on the contribution of innate immune cells on metastatic cell recruitment. Metastatic cell recruitment has been observed in immune-competent mice^17^, and this methodology could be applied to investigate the combined role of innate and adaptive arms of the immune response on homing. The innate immune cells used to generate the D-SCM in our study are recruited to a PLG scaffold following implantation. The local delivery of rHp from an inner layer in the scaffold was based on a previous report for enhancing survival of transplanted islets with localized delivery of trophic factors^40^. The release of haptoglobin from the PLG scaffold increased the recruitment of tumor cells to the scaffold. This *in vivo* result, coupled with our *in vitro* validation studies, is the first demonstration of haptoglobin mediating tumor cell homing to a defined niche, and implicates haptoglobin as a factor contributing to the spread of metastatic disease. The scaffold technology may also be utilized to evaluate the effects of additional factors contributing to metastatic disease, such as ECM proteins, in order to investigate organ-specific metastasis. Additionally, our haptoglobin scaffolds provide a synthetic pre-metastatic niche for capturing circulating tumor cells, which has implications in metastatic cancer detection, diagnosis, and therapy^17^.

Immune cells have been previously implicated in establishing a pre-metastatic niche that recruits cancer cells *in vivo*. Herein, we have developed a novel systems biology method to identify secreted factor involved in metastatic cell recruitment to the niche. Connecting secretome and TRACER data into a network allows for the identification of functional immune cell secreted factors that increase metastatic cell recruitment. After generating this network, we identified and validated haptoglobin as a critical mediator of homing. Additionally, an implantable biomaterial scaffold releasing haptoglobin attracted metastatic cells to the implant site, thus providing *in vivo* validation of our systems biology approach. Our systems biology technique allows for the identification of functional paracrine signaling factors within a secretome and is poised to uncover new candidates for targeted therapies against metastatic cell homing. More generally, connecting together secretomics and TRACER may be employed to identify secreted factors relevant for paracrine signaling that may underlie a variety of cell phenotypes.

## Materials and Methods

### MDA-MB-231 cell culture

The human breast adenocarcinoma cell line MDA-MB-231 was used for all *in vitro* experiments. MDA-MB-231 cells were routinely cultured on tissue culture polystyrene flasks in RPMI 1640 media supplemented with 10% fetal bovine serum (FBS), 1% penicillin-streptomycin solution, 1% non-essential amino acids, and 1% sodium pyruvate (Life Technologies). Media was exchanged every other day. Once ~80% confluent, cells were harvested with TrypLE Express (Life Technologies) solution and counted using a Trypan blue stain (Sigma Aldrich) and a Cell Countess automated hemocytometer (Life Technologies). All cells were cultured in a humidified 5% CO_2_ incubator at 37 °C.

### Transwell assays (invasion, migration, transendothelial migration)

For all assays, confluent cultures of MDA-MB-231 cells were serum starved overnight in serum free RPMI 1640 supplemented with 1% penicillin-streptomycin solution, 1% non-essential amino acids, and 1% sodium pyruvate prior to harvesting. Invasion assays were performed using Matrigel-coated transwell chambers with 8 μm pores (BD Biosciences). The membranes were hydrated with serum free media for 1 hour before plating cells. Serum-free cell suspensions were prepared and plated in transwell inserts at a density of 50,000 cells per insert, and the inserts were placed in RPMI and conditioned media supplemented with 2.0% FBS. Transwell inserts with cells were incubated for 24 hours in a humidified 5% CO_2_ incubator at 37 °C. After the incubation period, the cells on the top of the membrane were scraped away using a cotton swab soaked in PBS. Cells were fixed and stained in a 0.5% wt/vol crystal violet solution using a 60% EtOH/40% PBS solvent.

Migration assays were performed similarly using tissue culture treated transwell chambers with 8 μm pores (Corning). Serum-free cell suspensions were prepared and plated on transwells at a density of 50,000 cells per well, and the inserts were places in a separate 24 well plate containing RPMI and conditioned media supplemented with 0.2% FBS. Chambers were incubated for 24 hours and fixed with crystal violet.

For transendothelial migration assays, 8 μm pore transwell inserts (Corning) were coated with a 0.125 mg/mL solution of Matrigel (BD Biosciences) in serum-free RPMI for 1 hour at 37 °C. Human umbilical vein endothelial cells (HUVEC) were cultured in EndoGro-LS Complete Medium (Millipore), harvested with TrypLE, and plated in the transwell inserts at a density of 50,000 cells/well. HUVECs were allowed to attach overnight. The following day, serum-starved MDA-MB-231 cells labeled with mCherry were plated on top of the HUVECs at a density of 50,000 cells/well and incubated for 24 hours. Following incubation, cells inside the membrane were scraped away with a cotton swab soaked in PBS. Cells on the bottom of the membrane were fixed with 4% paraformaldehyde, and mounted on a glass cover slip with mounting media for fluorescence imaging.

For all assays, cells were imaged directly on the membrane with a Nikon Eclipse inverted microscope, and imaged at 10X. Four images per well were captured and cell numbers were quantified using ImageJ. All experiments were performed in triplicate.

### MTS proliferation assay

MDA-MB-231 cells were harvested and seeded at a density of 1000 cells/well in a 96 well plate. Cell proliferation was quantified using the CellTiter 96 Aqueous Non-Radioactive Proliferation Assay (Promega). Briefly, 3-(4,5-dimethylthiazol-2-yl)-5-(3-carbomethoxyphenyl)-2-(4-sulfophenyl)-2H-tetrazolium (MTS) and phenazine methosulfate (PMS) were combined and added to phenol red free RPMI media at a 1 to 5 dilution. The MTS solution was added to wells at daily time points for 4 days. After incubation for 4 hours, formazan levels were quantified using a Cytation3 plate reader (BioTek) at an absorbance wavelength of 490 nm. All formazan readings were normalized to the standard curve and to the initial time point. Statistical significance at each time point was determined using two-way ANOVA with *p* < 0.05. Error bars on all proliferation data represent the standard error of the sample mean.

### Splenocyte conditioned media preparation

Animal studies were performed in accordance with and approved by the Northwestern University Institutional Animal Care and Use Committee (IACUC). Immunodeficient NOD-scid IL2Rgamma^null^ (NSG) female mice (Jackson Labs) were injected with 2.0 × 10^6^ MDA-MB-231BR cells^41^ labeled with tdTomato and F-luciferase reporters in the right mammary fat pad.

Spleens were harvested 28 days later from tumor-bearing and tumor-free mice, ballooned using injections of 0.38 mg/mL solutions of liberase LT (Roche Diagnostics), and minced using micro-scissors. Minced tissue was incubated at 37 °C for 20 minutes, neutralized with 0.125 M EDTA, and processed into cell suspensions using FACS buffer (1X PBS, 0.5% BSA, 2 mM EDTA) and a 70 μm cell sieve. Cell suspensions were centrifuged using a swinging bucket rotor at 500 × g for 5 minutes at 4 °C. The supernatant was removed and the cells was re-suspended in ACK buffer (Life Technologies) for 2 minutes, neutralized with PBS and re-centrifuged. The splenocyte pellet was re-suspended in RPMI 1640 and cells were counted using a Trypan blue stain and a Cell Countess automated hemocytometer (Life Technologies). Cells were plated at 10^6^ cells/mL of serum free RPMI 1640 media supplemented with 1% penicillin-streptomycin, 1% non-essential amino acids, and 1% sodium pyruvate. All media was conditioned for 48 hours, filtered through a 0.22 μm filtration unit (Millipore), and stored at −80 °C until use.

Flow cytometry of leukocyte populations were performed as previously described^17^. Splenocytes were blocked with anti-CD16/32 (1:50, Biolegend) and stained for viability using fixable blue dead cell stain kit (Life Technologies). Cells were then stained with AlexaFluor700-conjugated anti-CD45 (30- F11, 1:100; BD Biosciences), Pacific Blue-conjugated anti-Gr-1 (RB6-8C5, 1:100; Biolegend), FITC-conjugated anti-Ly-6 C (HK1.4, 1:50; Biolegend), PE-Cyanine7-conjugated anti-F4/80 (BM8, 1:25; Biolegend), APC-conjugated anti-CD11c (N418, 1:40; eBioscience), and V500-conjugated anti-CD11b (M1/70, 1:100; eBioscience). For analysis, samples were re-suspended in FACS buffer and analyzed using a BD LSR Fortessa flow cytometer (Becton Dickinson Immunocytometry Systems, BDIS).

### Secretome analysis

Proteins in SCM samples were concentrated for secretomics analysis using a 3 kDa Amicon cellulose centrifugal filter unit (Millipore). For each concentrated conditioned media sample (three biological replicates), 5 μg of protein was solubilized by adding 8 M urea and incubating at 50 °C for 60 min. Following denaturation, proteins were solubilized and reduced by adding 10 mM DTT (final concentration 1 mM) and incubating at 50 °C for 15 min. After reduction, proteins were alkylated by adding 100 mM iodoacetamide (final concentration 10 mM) and incubated in the dark at room temperature for 15 min. Protein samples were digested by diluting the 8 M urea solution to 1 M by adding 100 mM ammonium bicarbonate and trypsin. Samples were digested at 37 °C overnight. The digested samples were desalted using reverse phase C18 spin columns (Thermo Fisher Scientific). After desalting, the peptides were concentrated *in vaccuo* until dry. After drying, peptides were suspended in 5% acetonitrile and 0.1% formic acid. The samples were loaded directly onto a 15 cm long, 75 μM reversed phase capillary column (ProteoPep™ II C18, 300 Å, 5 μm size, New Objective) and separated using a 200-minute gradient from 5% acetonitrile to 100% acetonitrile on a Proxeon Easy n-LC II (Thermo Scientific). The peptides were eluted into an LTQ Orbitrap Velos mass spectrometer (Thermo Scientific) with electrospray ionization at a 350 nL/minute flow rate. The mass spectrometer was operated in data dependent mode. For each MS1 precursor ion scan, the ten most intense ions were selected for fragmentation by CID (collision induced dissociation). Additional parameters for mass spectrometry analysis included setting the resolution of MS1 at 60,000, the normalized collision energy at 35%, the activation time at 10 ms, and the isolation width at 1.5. Charge states +4 and higher were rejected.

The data were processed using Proteome Discoverer (version 1.4, Thermo Scientific) and searched using an embedded SEQUEST HT search engine. The data were searched against a mouse reference proteome (September 2013, uniprot.org). Additional search parameters were as follows: (i) enzyme specificity: trypsin, (ii) fixed modification: cysteine carbamidomethylation, (iii) variable modification: methionine oxidation and N-terminal acetylation, (iv) precursor mass tolerance was ± 10 ppm, and (v) fragment ion mass tolerance was ± 0.8 Da. All the spectra were searched against target/decoy databases and results were used to estimate the q values with the Percolator algorithm embedded in Proteome discoverer 1.4. The peptide identification was considered valid at q value < 0.1 and were grouped for protein inference to satisfy the rule of parsimony. Further, each protein in the final identification list was considered valid if supported with a minimum of one unique peptide.

Proteins were quantified using spectral counting^42^ and normalized spectral abundance factors (NSAF)^43^,^44^. The NSAF normalization takes into consideration of the length of the protein, which may result into higher spectral count per protein. Initially, the total number of spectral counts (Spc) per protein was divided by the peptide length (L), and then divided by the sum (∑ spc/L) of all the values in the sample. Proteins were determined significantly changed if t-test on a significance level of 90% and if log2 fold change was greater than or equal to 1.5. The mass spectrometry data have been deposited to the ProteomeXchange Consortium^45^ via the PRIDE partner repository with the dataset identifier PXD002051.

### Western blotting

Concentrated SCM protein samples were resolved on a 10% SDS-PAGE gel and transferred to PVDF nitrocellulose membrane (BioRad). The membrane was blocked in a 5% dry milk solution for 1 hour, rinsed in 1X TBS-T solution, and incubated with rabbit anti-mouse polyclonal antibody against haptoglobin in a 5% dry milk solution overnight (1:250 dilution). After rinsing, a secondary antibody solution of anti-rabbit horseradish peroxidase (1:5000 dilution) was added and incubated for 1 hour. Haptoglobin protein was visualized via chemiluminescence and developed on film. Band density was quantified using Image J freeware.

### TRanscriptional Activity CEll aRray (TRACER)

Cell arrays were performed as previously described^16^,^27^,^46^,^47^. Harvested MDA-MB-231 cells were suspended in RPMI media to a final concentration of 50 cells/μL. 400 μL of this suspension was aliquoted into separate 1.5 mL Eppendorf tubes for viral infection. The aliquot was mixed with lentiviral vectors containing TF reporter constructs^16^ at a multiplicity of infection (MOI) of approximately 10 virions per cell. Cells and virus were mixed and plated at 2000 cells/well in a black, clear bottom, 384-well plate (Greiner Bio-One). Each TF reporter is represented with *n* = 4 measurements per array plate, and arrays were repeated a total of 6 times. After infection, cells were incubated for 48 hours.

To measure TF activity, D-luciferin (DLuc, RR Labs, Inc.) diluted in the appropriate media was added to wells in excess at a final concentration of 2 mM. After a 45-minute incubation period with the DLuc, the luminescence was quantified using an IVIS Lumina LTE imaging system (Caliper Life Sciences). Cells plated without virus infection served as negative controls for non-enzymatic DLuc degradation. A positive control consisted of a TA-FLuc reporter construct without any additional TF binding elements, which was used to determine basal promoter activity. Luminescence was quantified using an IVIS Lumina LTE imaging system (Caliper Life Sciences). All luminescence readings, measured in photon flux (photons/second), were normalized to the TA luminescence. On Day 0, cells were treated with either RPMI or D-SCM containing 2 mM of DLuc and 10% FBS. Bioluminescence imaging was conducted every 2 hours, and 5 reads were taken in one day. Each TF reporter is represented with *n* = 4 measurements per array plate, and arrays were repeated a total of 6 times.

Initial methodology to normalize and determine statistical significance was slightly modified^27^. Array data was log2 transformed and filtered to eliminate all intensities below background (*p* < 0.05). The background was defined as the mean measured intensity in non-infected cells subject to the same treatment at the same time and plate. At each time-point, the TA control reporter and the control condition were used to normalize reporter activity to calculate the fold-change between D-SCM vs. RPMI, rHp vs. RPMI, and HpAb vs. D-SCM, respectively. Normalized values that were identified to be outliers (*p* < 0.003) for each reporter were removed.

Normalized log2 TF activity fold-change of SCM, rHp, and HpAb were compared directly to control conditions using the limma package in R^48^. A linear model was fit to the normalized log2 values for each TF and was used to generate estimated coefficients and squared errors for each time point of the compared samples. The estimated coefficients and squared errors were then used to compute moderated t-statistics, moderated F-statistics, and log-odds of differential expression. Adjusted *p*-values were computed using the Benjamini-Hochberg procedure to correct for multiple comparisons^49^. TFs identified to be differentially active had an adjusted *p*-value of less than 0.05. To generate heat-maps, the replicate log2 fold-change for each condition and time-point was averaged. Normalized values were then clustered by *k*-means clustering with random starts. The sum-of-square error was computed for each cluster using the group mean. The optimal number of clusters was determined by maximizing the difference between the sum-of-square error of the computed *k*-means model and permuted null models.

### MetaCore analysis

All interaction networks were generated using MetaCore (Thomson Reuters). The network to predict downstream TFs included the 144 identified secreted factors in D-SCM and lists of all known human receptors and transcription factors obtained from MetaCore. In another network to determine functional interactions, we included the D-SCM secreted factors, a list of all known human receptors, and significantly active TFs from our TRACER screen. Secreted factors candidates were identified as ligands showing direct interactions with a receptor and downstream TFs. The TFs were expanded by one interaction to generate a list of downstream TFs.

### *In vitro* haptoglobin validation studies

Invasion, migration, transendothelial migration, MTS assays, and TRACER arrays were performed as described above. Recombinant haptoglobin (rHp, ProSpec) was diluted in serum-free RPMI at a concentration of 2 μg/mL. Haptoglobin antibody (HpAb, Abcam) was added to SCM at a dilution of 1:250, and the solution was incubated on ice for 30 min before use. For arrays, rHp and HpAb solutions were supplemented with 2 mM of DLuc and added prior to bioluminescence imaging.

### Layered protein scaffold fabrication and implantation

Layered PLG scaffolds containing 2 μg of rHp were fabricated as previously described^40^. Briefly, PLG microspheres for scaffolds were prepared by emulsifying 2% and 6% solution of PLG (Lakeshore Biomaterials; 75:25 lactide:glycolide, i.v. = 0.76 dL/g) in dicholoromethane in 1% poly(vinyl alcohol) (PVA). Microspheres were centrifuged and washed four times with deionized water to remove PVA and lyophilized overnight. For inner layers, 1 mg of 2% PLG microparticles was resuspended in a 1 mg/mL aqueous solution of D-mannitol. For haptoglobin scaffolds, 2% PLG particles were resuspended in a D-mannitol solution containing 2 μg of recombinant haptoglobin (Prospec). The PLG particles were flash frozen in liquid nitrogen and lyophilized overnight. The powder was pressed into 3 mm disks using a hand-press (Pike Technologies). Next, 6% PLG microspheres were mixed with 250–425 μm salt particles in a 1:33 ratio. The inner layers were pressed in between the 6% PLG-salt mixture in a steel die at 1500 psi. The layered scaffolds were gas-foamed and salt particles were leached in water for 30 minutes. Scaffolds were briefly sterilized with 70% ethanol and rinsed with water before surgical implantation. For SEM imaging, scaffolds were cut in half with a razor, mounted and pre-coated with 10 nm Au particles, and imaged using a high vacuum field-emission SEM (NOVA 600 NanoLab, FEI company) using a voltage of 20 kV and a current of 48 pA.

After performing orthotopic tumor inoculations in NSG mice, 2 scaffolds per mouse were implanted in the intraperitoneal fat pads 7 days post-tumor inoculation, and explanted 7 days post-implantation. TdTomato + MDA-MB-231BR cells were isolated from scaffolds using liberase LT (Roche) and analyzed using a BD LSR Fortessa flow cytometer (Becton Dickinson Immunocytometry Systems).

### Statistical significance

All data are shown as mean ± standard error (SEM) unless otherwise noted. Significance was claimed with *p*-values less than 0.05, determined using unpaired Student’s *t*-tests for single comparisons or one-way ANOVA with Bonferroni testing for multiple comparisons. The Fisher’s exact test was used to test the significance of overlap between the two TF lists. Statistical analysis was performed using GraphPad Prism.

## Additional Information

**How to cite this article**: Aguado, B. A. *et al.* Secretome identification of immune cell factors mediating metastatic cell homing. *Sci. Rep.* **5**, 17566; doi: 10.1038/srep17566 (2015).

## Supplementary Material

Supplementary Information
