# Supplementary material for: Secretome identification of immune cell factors mediating metastatic cell homing
Source: Sci Rep. 2015 Dec 4;5:17566. doi: 10.1038/srep17566 (PMC4669442; doi:10.1038/srep17566)
Supplement: Supplementary Information [file srep17566-s1.pdf]

SUPPLEMENTARY INFORMATION

Secretome identification of immune cell factors mediating metastatic cell homing

Brian A. Aguado, Jia J. Wu, Dhaval Nanavati, Samira M. Azarin, Shreyas S. Rao, Grace G. Bushnell, Chaitanya B. Medicherla, and Lonnie D. Shea

Inventory of Supplementary Information

Supplementary Figure 1

Supplementary Table 1

Supplementary Figure 2

Supplementary Figure 3

Supplementary Table 2

Supplementary Table 3

Supplementary Figure 4

Supplementary Figure 5

Supplementary Figure 6

Supplementary Figure 7

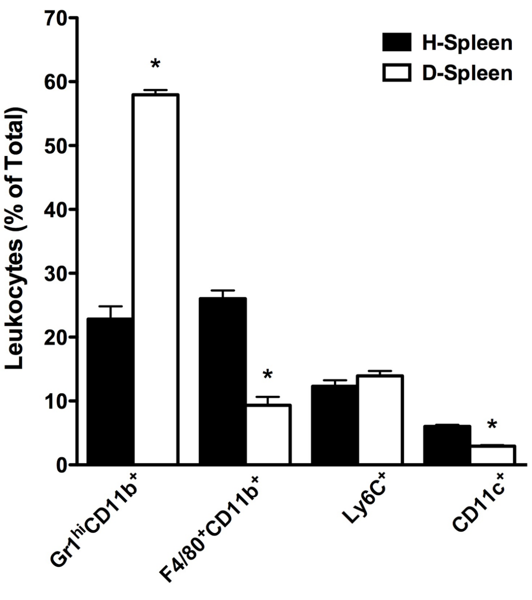

Supplementary Figure 1 - Flow cytometric analysis of healthy (H-) and diseased (D-) spleens (day 28 post-inoculation) from NSG mice. Results are shown as percentage of $Gr1^{hi}CD11b^{+}$, $F4/80^{+}CD11b^{+}$, $Ly6C^{+}$, or $CD11c^{+}$ cells in the total population of $CD45^{+}$ leukocytes. Error bars denote SEM ($n = 8$, $*p < 0.001$ compared to healthy spleen).

Accession	Description	MW [kDa]	Length	#PSM - D-SCM 1	#PSM - D-SCM 2	#PSM - D-SCM 3	Average D-SCM	#PSM - H-SCM 1	#PSM - H-SCM 2	#PSM - H-SCM 3	Average H-SCM	Log 2 Fold Change	P-Value
P11247	Myeloperoxidase OS=Mus musculus GN=Mpo PE=2 SV=2 - [PERM_MOUSE]	81.1	718	37	28	52	39.00	1	1	4	2.00	4.3	0.026
Q9ET01	Glycogen phosphorylase, liver form OS=Mus musculus GN=Pygl PE=1 SV=4 - [PYGL_MOUSE]	97.4	850	44	48	49	47.00	8	10	6	8.00	2.6	0.000
P05201	Aspartate aminotransferase, cytoplasmic OS=Mus musculus GN=Got1 PE=1 SV=3 - [AATC_MOUSE]	46.2	413	3	8	8	6.33	1	2	1	1.33	2.2	0.042
Q9D154	Leukocyte elastase inhibitor A OS=Mus musculus GN=Serpinb1a PE=1 SV=1 - [ILEUA_MOUSE]	42.5	379	67	65	65	65.67		18	11	14.50	2.2	0.000
P30681	High mobility group protein B2 OS=Mus musculus GN=Hmgb2 PE=1 SV=3 - [HMGB2_MOUSE]	24.1	210	33	42	33	36.00	3	9	13	8.33	2.1	0.003
E9Q3W4	Plectin OS=Mus musculus GN=Plec PE=4 SV=1 - [E9Q3W4_MOUSE]	498.8	4386	9	9	9	9.00	1	1	5	2.33	1.9	0.007
Q9R111	Guanine deaminase OS=Mus musculus GN=Gda PE=1 SV=1 - [GUAD_MOUSE]	51.0	454	25	24	30	26.33		9	5	7.00	1.9	0.006
O88593	Peptidoglycan recognition protein 1 OS=Mus musculus GN=Pglyrp1 PE=2 SV=1 - [PGRP1_MOUSE]	20.5	182	18	16	22	18.67	4		6	5.00	1.9	0.011
P41245	Matrix metalloproteinase-9 OS=Mus musculus GN=Mmp9 PE=2 SV=2 - [MMP9_MOUSE]	80.5	730	17	22	16	18.33		4	6	5.00	1.9	0.013
P29351	Tyrosine-protein phosphatase non-receptor type 6 OS=Mus musculus GN=Ptpn6 PE=1 SV=2 - [PTN6_MOUSE]	67.5	595	7	4	7	6.00	1	3	1	1.67	1.8	0.023
Q9QUH0	Glutaredoxin-1 OS=Mus musculus GN=Glrx PE=1 SV=3 - [GLRX1_MOUSE]	11.9	107	8	4	6	6.00	1	3	1	1.67	1.8	0.031
D3Z2P2	Chitinase-3-like protein 1 OS=Mus musculus GN=Chi3l1 PE=2 SV=1 - [D3Z2P2_MOUSE]	42.8	389	19	16	14	16.33	4	4	6	4.67	1.8	0.002
Q91Z98	Chitinase-3-like protein 4 OS=Mus musculus GN=Chi3l4 PE=1 SV=2 - [CH3L4_MOUSE]	44.9	402	19	19	20	19.33	1	15	1	5.67	1.8	0.043
Q00612	Glucose-6-phosphate 1-dehydrogenase X OS=Mus musculus GN=G6pdx PE=1 SV=3 - [G6PD1_MOUSE]	59.2	515	44	37	50	43.67	11	15	13	13.00	1.7	0.001
Q3TRM8	Hexokinase-3 OS=Mus musculus GN=Hk3 PE=1 SV=2 - [HXK3_MOUSE]	100.0	922	17	12	25	18.00	9	5	3	5.67	1.7	0.042
P31786	Acyl-CoA-binding protein OS=Mus musculus GN=Dbi PE=1 SV=2 - [ACBP_MOUSE]	10.0	87	6	6	10	7.33	1	5	1	2.33	1.7	0.057
Q91YR9	Prostaglandin reductase 1 OS=Mus musculus GN=Ptgr1 PE=2 SV=2 - [PTGR1_MOUSE]	35.5	329	14	12	16	14.00	3		6	4.50	1.6	0.015
A3KGU5	Spectrin alpha chain, non-erythrocytic 1 OS=Mus musculus GN=Sptan1 PE=2 SV=1 - [A3KGU5_MOUSE]	282.7	2457	10	7	6	7.67	2		3	2.50	1.6	0.048
P01027	Complement C3 OS=Mus musculus GN=C3 PE=1 SV=3 - [CO3_MOUSE]	186.4	1663	27	17	26	23.33	5	11	7	7.67	1.6	0.013
Q61166	Microtubule-associated protein RP/EB 1 OS=Mus musculus GN=Mapre1 PE=1 SV=3 - [MARE1_MOUSE]	30.0	268	7	7	10	8.00	1	1	6	2.67	1.6	0.052
P24527	Leukotriene A-4 hydrolase OS=Mus musculus GN=Lta4h PE=1 SV=4 - [LKHA4_MOUSE]	69.0	611	51	54	68	57.67	15	22	21	19.33	1.6	0.003
Q61646	Haptoglobin OS=Mus musculus GN=Hp PE=1 SV=1 - [HPT_MOUSE]	38.7	347	22	19	23	21.33		6	9	7.50	1.5	0.005
Q9DCD0	6-phosphogluconate dehydrogenase, decarboxylating OS=Mus musculus GN=Pgd PE=2 SV=3 - [6PGD_MOUSE]	53.2	483	53	43	53	49.67	9	23	22	18.00	1.5	0.005
P08071	Lactotransferrin OS=Mus musculus GN=Ltf PE=2 SV=4 - [TRFL_MOUSE]	77.8	707	125	128	129	127.33	22	58	75	51.67	1.3	0.008
Q921M7	Protein FAM49B OS=Mus musculus GN=Fam49b PE=2 SV=1 - [FA49B_MOUSE]	36.8	324	7	6	11	8.00	2	5	3	3.33	1.3	0.057
Q922B2	Aspartate--tRNA ligase, cytoplasmic OS=Mus musculus GN=Dars PE=2 SV=2 - [SYDC_MOUSE]	57.1	501	3	4	5	4.00	1	1	3	1.67	1.3	0.057
P06745	Glucose-6-phosphate isomerase OS=Mus musculus GN=Gpi PE=1 SV=4 - [G6PI_MOUSE]	62.7	558	74	76	76	75.33	27	31	38	32.00	1.2	0.000
P10649	Glutathione S-transferase Mu 1 OS=Mus musculus GN=Gstm1 PE=1 SV=2 - [GSTM1_MOUSE]	26.0	218	27	22	28	25.67	8	11	14	11.00	1.2	0.004
Q60865	Caprin-1 OS=Mus musculus GN=Caprin1 PE=1 SV=2 - [CAPR1_MOUSE]	78.1	707	5	4	5	4.67	1	4	1	2.00	1.2	0.065
P07356	Annexin A2 OS=Mus musculus GN=Anxa2 PE=1 SV=2 - [ANXA2_MOUSE]	38.7	339	13	22	16	17.00	6	10	6	7.33	1.2	0.031
Q9R0P5	Destrin OS=Mus musculus GN=Dstn PE=1 SV=3 - [DEST_MOUSE]	18.5	165	6	8	10	8.00	3		4	3.50	1.2	0.061
P05064	Fructose-bisphosphate aldolase A OS=Mus musculus GN=Aldoa PE=1 SV=2 - [ALDOA_MOUSE]	39.3	364	21	33	33	29.00	11	15	13	13.00	1.2	0.018
P47791	Glutathione reductase, mitochondrial OS=Mus musculus GN=Gsr PE=2 SV=3 - [GSHR_MOUSE]	53.6	500	15	15	19	16.33	11	5	6	7.33	1.2	0.017
Q9R1P1	Proteasome subunit beta type-3 OS=Mus musculus GN=Psmb3 PE=1 SV=1 - [PSB3_MOUSE]	22.9	205	10	9	11	10.00	5	5	4	4.67	1.1	0.001
P52480	Pyruvate kinase PKM OS=Mus musculus GN=Pkm PE=1 SV=4 - [KPYM_MOUSE]	57.8	531	36	36	39	37.00	8	20	24	17.33	1.1	0.016
P63028	Translationally-controlled tumor protein OS=Mus musculus GN=Tpt1 PE=1 SV=1 - [TCTP_MOUSE]	19.4	172	14	19	16	16.33	6	7	10	7.67	1.1	0.010
Q9Z0P5	Twinfilin-2 OS=Mus musculus GN=Twf2 PE=1 SV=1 - [TWF2_MOUSE]	39.4	349	6	6	7	6.33		3	3	3.00	1.1	0.004
Q61599	Rho GDP-dissociation inhibitor 2 OS=Mus musculus GN=Arhgdib PE=1 SV=3 - [GDIR2_MOUSE]	22.8	200	31	39	35	35.00	11	21	19	17.00	1.0	0.009
Q60864	Stress-induced-phosphoprotein 1 OS=Mus musculus GN=Stip1 PE=1 SV=1 - [STIP1_MOUSE]	62.5	543	9	8	10	9.00		4	5	4.50	1.0	0.012
P13020	Gelsolin OS=Mus musculus GN=Gsn PE=1 SV=3 - [GELS_MOUSE]	85.9	780	10	9	11	10.00		6	4	5.00	1.0	0.018
G3UVV4	Hexokinase 1, isoform CRA_f OS=Mus musculus GN=Hk1 PE=3 SV=1 - [G3UVV4_MOUSE]	101.8	917	6	7	8	7.00	3	4		3.50	1.0	0.025
P11672	Neutrophil gelatinase-associated lipocalin OS=Mus musculus GN=Lcn2 PE=1 SV=1 - [NGAL_MOUSE]	22.9	200	26	20	20	22.00	5	13	15	11.00	1.0	0.160
Q9JKF1	Ras GTPase-activating-like protein IQGAP1 OS=Mus musculus GN=Iqgap1 PE=1 SV=2 - [IQGA1_MOUSE]	188.6	1657	24	22	31	25.67	9	17	13	13.00	1.0	0.024
P40142	Transketolase OS=Mus musculus GN=Tkt PE=1 SV=1 - [TKT_MOUSE]	67.6	623	85	86	93	88.00	34	53	49	45.33	1.0	0.002
O89053	Coronin-1A OS=Mus musculus GN=Coro1a PE=1 SV=5 - [COR1A_MOUSE]	51.0	461	36	35	31	34.00	14	17	22	17.67	0.9	0.004
B7FAU9	Filamin, alpha OS=Mus musculus GN=Flna PE=4 SV=1 - [B7FAU9_MOUSE]	280.3	2639	96	93	98	95.67	28	58	65	50.33	0.9	0.017
P08905	Lysozyme C-2 OS=Mus musculus GN=Lyz2 PE=1 SV=2 - [LYZ2_MOUSE]	16.7	148	20	18	25	21.00	11	11	13	11.67	0.8	0.013
Q9DBJ1	Phosphoglycerate mutase 1 OS=Mus musculus GN=Pgam1 PE=1 SV=3 - [PGAM1_MOUSE]	28.8	254	28	39	32	33.00	14	18	23	18.33	0.8	0.024
P10810	Monocyte differentiation antigen CD14 OS=Mus musculus GN=Cd14 PE=1 SV=1 - [CD14_MOUSE]	39.2	366	10	10	14	11.33	6	7	6	6.33	0.8	0.022
P31725	Protein S100-A9 OS=Mus musculus GN=S100a9 PE=1 SV=3 - [S10A9_MOUSE]	13.0	113	44	32	31	35.67	14	25	21	20.00	0.8	0.041
P14211	Calreticulin OS=Mus musculus GN=Calr PE=1 SV=1 - [CALR_MOUSE]	48.0	416	21	28	27	25.33	20	10	13	14.33	0.8	0.040
Q9CQ60	6-phosphogluconolactonase OS=Mus musculus GN=Pgls PE=2 SV=1 - [6PGL_MOUSE]	27.2	257	15	14	14	14.33	6	11	8	8.33	0.8	0.016
Q9CR16	Peptidyl-prolyl cis-trans isomerase D OS=Mus musculus GN=Ppid PE=1 SV=3 - [PPID_MOUSE]	40.7	370	5	6	7	6.00		3	4	3.50	0.8	0.058
Q9CVB6	Actin-related protein 2/3 complex subunit 2 OS=Mus musculus GN=Arpc2 PE=1 SV=3 - [ARPC2_MOUSE]	34.3	300	29	24	27	26.67	14	22	11	15.67	0.8	0.038
Q00519	Xanthine dehydrogenase/oxidase OS=Mus musculus GN=Xdh PE=1 SV=5 - [XDH_MOUSE]	146.5	1335	17	16	16	16.33	15	8	7	10.00	0.7	0.067
Q91VW3	SH3 domain-binding glutamic protein 3 OS=Mus musculus GN=Sh3bgrl3 PE=1 SV=1 - [SH3L3_MOUSE]	10.5	93	6	7	8	7.00	3	5	5	4.33	0.7	0.039
P17742	Peptidyl-prolyl cis-trans isomerase A OS=Mus musculus GN=Ppia PE=1 SV=2 - [PPIA_MOUSE]	18.0	164	33	31	34	32.67	22	17	22	20.33	0.7	0.003
P27005	Protein S100-A8 OS=Mus musculus GN=S100a8 PE=1 SV=3 - [S10A8_MOUSE]	10.3	89	77	89	84	83.33	32	71	57	53.33	0.6	0.066
P68254	14-3-3 protein theta OS=Mus musculus GN=Ywhaq PE=1 SV=1 - [1433T_MOUSE]	27.8	245	15	13	17	15.00	11	9	9	9.67	0.6	0.016
P68037	Ubiquitin-conjugating enzyme E2 L3 OS=Mus musculus GN=Ube2l3 PE=2 SV=1 - [UB2L3_MOUSE]	17.9	154	6	5	6	5.67	3	5	3	3.67	0.6	0.055
H7BWZ3	Actin-related protein 2/3 complex subunit 3 OS=Mus musculus GN=Arpc3 PE=2 SV=1 - [H7BWZ3_MOUSE]	19.6	170	7	8	8	7.67	6	5	4	5.00	0.6	0.016
O35744	Chitinase-3-like protein 3 OS=Mus musculus GN=Chi3l3 PE=1 SV=2 - [CH3L3_MOUSE]	44.4	398	62	59	68	63.00	33	42	50	41.67	0.6	0.019
P17182	Alpha-enolase OS=Mus musculus GN=Eno1 PE=1 SV=3 - [ENOA_MOUSE]	47.1	434	56	44	48	49.33	27	33	38	32.67	0.6	0.025
P16110	Galectin-3 OS=Mus musculus GN=Lgals3 PE=1 SV=3 - [LEG3_MOUSE]	27.5	264	27	24	23	24.67	10	20	19	16.33	0.6	0.070

Supplementary Table 1 - Summary of identified proteins from secretomics analysis of H-SCM and D-SCM. List of 144 protein matches identified as secreted proteins (115 identified in both D-SCM and H-SCM and 29 identified exclusively in D-SCM), with significance cut-off of $p < 0.1$. Matched proteins highlighted in green have peptide spectral matches with a log2 fold difference greater than 1.5 in D-SCM. Matched proteins highlighted in red have peptide spectral matches with a log2 fold difference greater than 1.5 in H-SCM.

Accession	Description	MW [kDa]	Length	#PSM - D-SCM 1	#PSM - D-SCM 2	#PSM - D-SCM 3	Average D-SCM	#PSM - H-SCM 1	#PSM - H-SCM 2	#PSM - H-SCM 3	Average H-SCM	Log 2 Fold Change	P-Value
P07901	Heat shock protein HSP 90-alpha OS=Mus musculus GN=Hsp90aa1 PE=1 SV=4 - [HS90A_MOUSE]	84.7	733	44	49	57	50.00	28	38	36	34.00	0.6	0.030
O08692	Myeloid bactenecin (F1) OS=Mus musculus GN=Ngp PE=2 SV=1 - [O08692_MOUSE]	19.3	167	22	21	23	22.00	11	16	18	15.00	0.6	0.032
P27773	Protein disulfide-isomerase A3 OS=Mus musculus GN=Pdia3 PE=1 SV=2 - [PDIA3_MOUSE]	56.6	505	43	53	52	49.33	30	37	36	34.33	0.5	0.018
P26040	Ezrin OS=Mus musculus GN=Ezr PE=1 SV=3 - [EZRI_MOUSE]	69.4	586	19		20	19.50	14	14		14.00	0.5	0.008
Q61233	Plastin-2 OS=Mus musculus GN=Lcp1 PE=1 SV=4 - [PLSL_MOUSE]	70.1	627	106	91	118	105.00	81	81	77	79.67	0.4	0.033
P63101	14-3-3 protein zeta/delta OS=Mus musculus GN=Ywhaz PE=1 SV=1 - [1433Z_MOUSE]	27.8	245	40	40	36	38.67	30	33	30	31.00	0.3	0.010
P20029	78 kDa glucose-regulated protein OS=Mus musculus GN=Hspa5 PE=1 SV=3 - [GRP78_MOUSE]	72.4	655	48	47	52	49.00	36	44	44	41.33	0.2	0.067
P27661	Histone H2AX OS=Mus musculus GN=H2afx PE=1 SV=2 - [H2AX_MOUSE]	15.1	143	16	16	17	16.33		19	19	19.00	-0.2	0.008
P08228	Superoxide dismutase [Cu-Zn] OS=Mus musculus GN=Sod1 PE=1 SV=2 - [SODC_MOUSE]	15.9	154	13	12	14	13.00	18	15	15	16.00	-0.3	0.060
P08249	Malate dehydrogenase, mitochondrial OS=Mus musculus GN=Mdh2 PE=1 SV=3 - [MDHM_MOUSE]	35.6	338	18	18	19	18.33	22	24	23	23.00	-0.3	0.002
P68372	Tubulin beta-4B chain OS=Mus musculus GN=Tubb4b PE=1 SV=1 - [TBB4B_MOUSE]	49.8	445	25	19		22.00	34	33	29	32.00	-0.5	0.044
Q61029	Lamina-associated polypeptide 2 OS=Mus musculus GN=Tmpo PE=1 SV=4 - [LAP2B_MOUSE]	50.3	452	8	9	9	8.67	10	14	14	12.67	-0.5	0.044
P43275	Histone H1.1 OS=Mus musculus GN=Hist1h1a PE=1 SV=2 - [H11_MOUSE]	21.8	213	8	8	8	8.00		11	13	12.00	-0.6	0.013
A1BN54	Alpha actinin 1a OS=Mus musculus GN=Actn1 PE=2 SV=1 - [A1BN54_MOUSE]	102.7	887	52	50	68	56.67	79	85	91	85.00	-0.6	0.013
Q8VCI0	Phospholipase B-like 1 OS=Mus musculus GN=Plbd1 PE=1 SV=1 - [PLBL1_MOUSE]	63.0	550	13	15	13	13.67	21	26	17	21.33	-0.6	0.046
A8DUK4	Beta-globin OS=Mus musculus GN=Hbb-b1 PE=3 SV=1 - [A8DUK4_MOUSE]	15.7	147	39	36	50	41.67	70	69	60	66.33	-0.7	0.010
Q9DCJ9	N-acetylneuraminate lyase OS=Mus musculus GN=Npl PE=1 SV=1 - [NPL_MOUSE]	35.1	320	7	6	5	6.00	9	13	10	10.67	-0.8	0.025
P47754	F-actin-capping protein subunit alpha-2 OS=Mus musculus GN=Capza2 PE=1 SV=3 - [CAZA2_MOUSE]	32.9	286	3	3	4	3.33	6	5	7	6.00	-0.8	0.016
Q9Z2X1	Heterogeneous nuclear ribonucleoprotein F OS=Mus musculus GN=Hnrnpf PE=1 SV=3 - [HNRPF_MOUSE]	45.7	415	5	7	4	5.33	8	10	11	9.67	-0.9	0.025
Q9JII6	Alcohol dehydrogenase [NADP(+)] OS=Mus musculus GN=Akr1a1 PE=1 SV=3 - [AK1A1_MOUSE]	36.6	325	7	4	7	6.00	10	14	9	11.00	-0.9	0.052
P14152	Malate dehydrogenase, cytoplasmic OS=Mus musculus GN=Mdh1 PE=1 SV=3 - [MDHC_MOUSE]	36.5	334	6	5	8	6.33	10	12	13	11.67	-0.9	0.013
P28076	Proteasome subunit beta type-9 OS=Mus musculus GN=Psmb9 PE=1 SV=1 - [PSB9_MOUSE]	23.4	219	4		3	3.50	6	7		6.50	-0.9	0.051
Q99LT0	Protein dpy-30 homolog OS=Mus musculus GN=Dpy30 PE=1 SV=1 - [DPY30_MOUSE]	11.2	99	4	4	5	4.33	8	9	8	8.33	-0.9	0.001
P35700	Peroxiredoxin-1 OS=Mus musculus GN=Prdx1 PE=1 SV=1 - [PRDX1_MOUSE]	22.2	199	13	13	16	14.00	29	25	27	27.00	-0.9	0.001
Q9R257	Heme-binding protein 1 OS=Mus musculus GN=Hebp1 PE=1 SV=2 - [HEBP1_MOUSE]	21.1	190	7	6	8	7.00	11	14	17	14.00	-1.0	0.019
P18242	Cathepsin D OS=Mus musculus GN=Ctsd PE=1 SV=1 - [CATD_MOUSE]	44.9	410	17	10	12	13.00	32	24	23	26.33	-1.0	0.019
P14733	Lamin-B1 OS=Mus musculus GN=Lmnb1 PE=1 SV=3 - [LMNB1_MOUSE]	66.7	588	10	21	16	15.67	23	36	38	32.33	-1.0	0.043
O88844	Isocitrate dehydrogenase [NADP] cytoplasmic OS=Mus musculus GN=Idh1 PE=1 SV=2 - [IDHC_MOUSE]	46.6	414	6	5	4	5.00	11	13	9	11.00	-1.1	0.010
Q8BG07	Phospholipase D4 OS=Mus musculus GN=Pld4 PE=2 SV=1 - [PLD4_MOUSE]	56.1	503	14	12	8	11.33	29	24	25	26.00	-1.2	0.003
P29416	Beta-hexosaminidase subunit alpha OS=Mus musculus GN=Hexa PE=2 SV=2 - [HEXA_MOUSE]	60.6	528	7		6	6.50	14	16	15	15.00	-1.2	0.002
H3BKM0	AP-2 complex subunit beta OS=Mus musculus GN=Ap2b1 PE=2 SV=1 - [H3BKM0_MOUSE]	101.3	913	5	2	5	4.00	12	9	7	9.33	-1.2	0.039
Q923D2	Flavin reductase (NADPH) OS=Mus musculus GN=Blvrb PE=2 SV=3 - [BLVRB_MOUSE]	22.2	206	8	6	8	7.33	21	19	16	18.67	-1.3	0.002
P21619	Lamin-B2 OS=Mus musculus GN=Lmnb2 PE=1 SV=2 - [LMNB2_MOUSE]	67.3	596		4	3	3.50		8	10	9.00	-1.4	0.039
P14901	Heme oxygenase 1 OS=Mus musculus GN=Hmox1 PE=1 SV=1 - [HMOX1_MOUSE]	32.9	289	9	10	9	9.33	23	26	25	24.67	-1.4	0.000
P20060	Beta-hexosaminidase subunit beta OS=Mus musculus GN=Hexb PE=2 SV=2 - [HEXB_MOUSE]	61.1	536	3	7	6	5.33	15	14	14	14.33	-1.4	0.002
Q9CPX4	Ferritin OS=Mus musculus GN=Ftl1 PE=2 SV=1 - [Q9CPX4_MOUSE]	20.8	183	32	32	31	31.67	96	107	58	87.00	-1.5	0.020
P09528	Ferritin heavy chain OS=Mus musculus GN=Fth1 PE=1 SV=2 - [FRIH_MOUSE]	21.1	182	14	18	12	14.67	38	40	43	40.33	-1.5	0.000
Q9QZQ8	Core histone macro-H2A.1 OS=Mus musculus GN=H2afy PE=1 SV=3 - [H2AY_MOUSE]	39.7	372	4	6	4	4.67		14	16	15.00	-1.7	0.003
P16675	Lysosomal protective protein OS=Mus musculus GN=Ctsa PE=1 SV=1 - [PPGB_MOUSE]	53.8	474	4	4		4.00	14	14	12	13.33	-1.7	0.002
P99024	Tubulin beta-5 chain OS=Mus musculus GN=Tubb5 PE=1 SV=1 - [TBB5_MOUSE]	49.6	444	1	1	28	10.00	34	35	33	34.00	-1.8	0.056
Q8R1G2	Carboxymethylenebutenolidase homolog OS=Mus musculus GN=Cmbl PE=2 SV=1 - [CMBL_MOUSE]	27.9	245	3	1	1	1.67	6	7	6	6.33	-1.9	0.003
P10605	Cathepsin B OS=Mus musculus GN=Ctsb PE=1 SV=2 - [CATB_MOUSE]	37.3	339	11	7	10	9.33	43	35	35	37.67	-2.0	0.001
Q9CY64	Biliverdin reductase A OS=Mus musculus GN=Blvra PE=2 SV=1 - [BIEA_MOUSE]	33.5	295	4	3	3	3.33	17	12	13	14.00	-2.1	0.002
Q9QZU3	Platelet glycoprotein V (Fragment) OS=Mus musculus GN=Gp5 PE=2 SV=1 - [Q9QZU3_MOUSE]	63.3	567	4	1	1	2.00	12	7	7	8.67	-2.1	0.027
P36552	Coproporphyrinogen-III oxidase, mitochondrial OS=Mus musculus GN=Cpox PE=1 SV=2 - [HEM6_MOUSE]	49.7	443	6	3	5	4.67	18	25	24	22.33	-2.3	0.002
Q9QWR8	Alpha-N-acetylgalactosaminidase OS=Mus musculus GN=Naga PE=2 SV=2 - [NAGAB_MOUSE]	47.2	415	4	4	1	3.00	18	18	18	18.00	-2.6	0.000
Q9WV54	Acid ceramidase OS=Mus musculus GN=Asah1 PE=1 SV=1 - [ASAH1_MOUSE]	44.6	394	6	1	1	2.67	19	17	13	16.33	-2.6	0.005
P15508	Spectrin beta chain, erythrocytic OS=Mus musculus GN=Sptb PE=1 SV=4 - [SPTB1_MOUSE]	245.1	2128	1	1	3	1.67	7	11	14	10.67	-2.7	0.014
P35441	Thrombospondin-1 OS=Mus musculus GN=Thbs1 PE=1 SV=1 - [TSP1_MOUSE]	129.6	1170	3	2		2.50	11	16	25	17.33	-2.8	0.068
Q62351	Transferrin receptor protein 1 OS=Mus musculus GN=Tfrc PE=1 SV=1 - [TFR1_MOUSE]	85.7	763	4	1	1	2.00	6	20	24	16.67	-3.1	0.057
P48678	Prelamin-A/C OS=Mus musculus GN=Lmna PE=1 SV=2 - [LMNA_MOUSE]	74.2	665	1	1	4	2.00	19	28	39	28.67	-3.8	0.010

Supplementary Table 1, Continued

Continuation of 115 secreted proteins identified in both H-SCM and D-SCM.

Accession	Description	MW [kDa]	#PSM - D-SCM 1	#PSM - D-SCM 2	#PSM - D-SCM 3	Average D-SCM
P00688	Pancreatic alpha-amylase OS=Mus musculus GN=Amy2 PE=1 SV=2 - [AMYP_MOUSE]	57.3	75	24	26	41.67
P08032	Spectrin alpha chain, erythrocytic 1 OS=Mus musculus GN=Spta1 PE=2 SV=3 - [SPTA1_MOUSE]	279.7	9	18	18	15.00
P00687	Alpha-amylase 1 OS=Mus musculus GN=Amy1 PE=1 SV=2 - [AMY1_MOUSE]	57.6	49	18	16	27.67
P63038	60 kDa heat shock protein, mitochondrial OS=Mus musculus GN=Hspd1 PE=1 SV=1 - [CH60_MOUSE]	60.9	5	12	12	9.67
O35405	Phospholipase D3 OS=Mus musculus GN=Pld3 PE=2 SV=1 - [PLD3_MOUSE]	54.4	14	12	11	12.33
P01899	H-2 class I histocompatibility antigen, D-B alpha chain OS=Mus musculus GN=H2-D1 PE=1 SV=2 - [HA11_MOUSE]	40.8	4	6	10	6.67
P21981	Protein-glutamine gamma-glutamyltransferase 2 OS=Mus musculus GN=Tgm2 PE=1 SV=4 - [TGM2_MOUSE]	77.0	10	8	9	9.00
Q9EQI5	Chemokine (C-X-C motif) ligand 7, isoform CRA_b OS=Mus musculus GN=Ppbp PE=4 SV=1 - [Q9EQI5_MOUSE]	12.2	6	5	9	6.67
Q9WVJ3	Carboxypeptidase Q OS=Mus musculus GN=Cpq PE=2 SV=1 - [CBPQ_MOUSE]	51.8	10	10	8	9.33
P05208	Chymotrypsin-like elastase family member 2A OS=Mus musculus GN=Cela2a PE=2 SV=1 - [CEL2A_MOUSE]	28.9	28	7	8	14.33
Q05117	Tartrate-resistant acid phosphatase type 5 OS=Mus musculus GN=Acp5 PE=2 SV=2 - [PPA5_MOUSE]	36.8	6	7	8	7.00
G5E850	Cytochrome b-5, isoform CRA_a OS=Mus musculus GN=Cyb5 PE=3 SV=1 - [G5E850_MOUSE]	11.1	4	3	7	4.67
Q9D1A2	Cytosolic non-specific dipeptidase OS=Mus musculus GN=Cndp2 PE=1 SV=1 - [CNDP2_MOUSE]	52.7	3	9	6	6.00
P45376	Aldose reductase OS=Mus musculus GN=Akr1b1 PE=1 SV=3 - [ALDR_MOUSE]	35.7	5	7	6	6.00
Q9R0T7	MCG15085 OS=Mus musculus GN=Try4 PE=2 SV=1 - [Q9R0T7_MOUSE]	26.3	11	6	6	7.67
Q7TPZ8	Carboxypeptidase A1 OS=Mus musculus GN=Cpa1 PE=2 SV=1 - [CBPA1_MOUSE]	47.4	33	5	6	14.67
Q91X79	Chymotrypsin-like elastase family member 1 OS=Mus musculus GN=Cela1 PE=2 SV=1 - [CELA1_MOUSE]	28.9	29	4	6	13.00
P48036	Annexin A5 OS=Mus musculus GN=Anxa5 PE=1 SV=1 - [ANXA5_MOUSE]	35.7	12	9	5	8.67
O89023	Tripeptidyl-peptidase 1 OS=Mus musculus GN=Tpp1 PE=1 SV=2 - [TPP1_MOUSE]	61.3	6	6	5	5.67
Q04447	Creatine kinase B-type OS=Mus musculus GN=Ckb PE=1 SV=1 - [KCRB_MOUSE]	42.7	6	5	5	5.33
P04117	Fatty acid-binding protein, adipocyte OS=Mus musculus GN=Fabp4 PE=1 SV=3 - [FABP4_MOUSE]	14.6	3	6	4	4.33
P13707	Glycerol-3-phosphate dehydrogenase [NAD(+)], cytoplasmic OS=Mus musculus GN=Gpd1 PE=1 SV=3 - [GPDA_MOUSE]	37.5	6	4	4	4.67
P49935	Pro-cathepsin H OS=Mus musculus GN=Ctsh PE=2 SV=2 - [CATH_MOUSE]	37.1	5	4	4	4.33
P56391	Cytochrome c oxidase subunit 6B1 OS=Mus musculus GN=Cox6b1 PE=1 SV=2 - [CX6B1_MOUSE]	10.1	4	4	4	4.00
D3Z7R1	Pyridoxal kinase OS=Mus musculus GN=Pdxk PE=2 SV=1 - [D3Z7R1_MOUSE]	30.2	4	4	4	4.00
Q61830	Macrophage mannose receptor 1 OS=Mus musculus GN=Mrc1 PE=1 SV=2 - [MRC1_MOUSE]	164.9	7	8	3	6.00
O35215	D-dopachrome decarboxylase OS=Mus musculus GN=Ddt PE=1 SV=3 - [DOPD_MOUSE]	13.1	6	4	3	4.33
O88958	Glucosamine-6-phosphate isomerase 1 OS=Mus musculus GN=Gnpda1 PE=2 SV=3 - [GNPI1_MOUSE]	32.5	4	4	3	3.67
Q3TCN2	Putative phospholipase B-like 2 OS=Mus musculus GN=Plbd2 PE=1 SV=2 - [PLBL2_MOUSE]	66.2	9	3	3	5.00

Supplementary Table 1, Continued

List of 29 secreted factors identified exclusively in D-SCM.

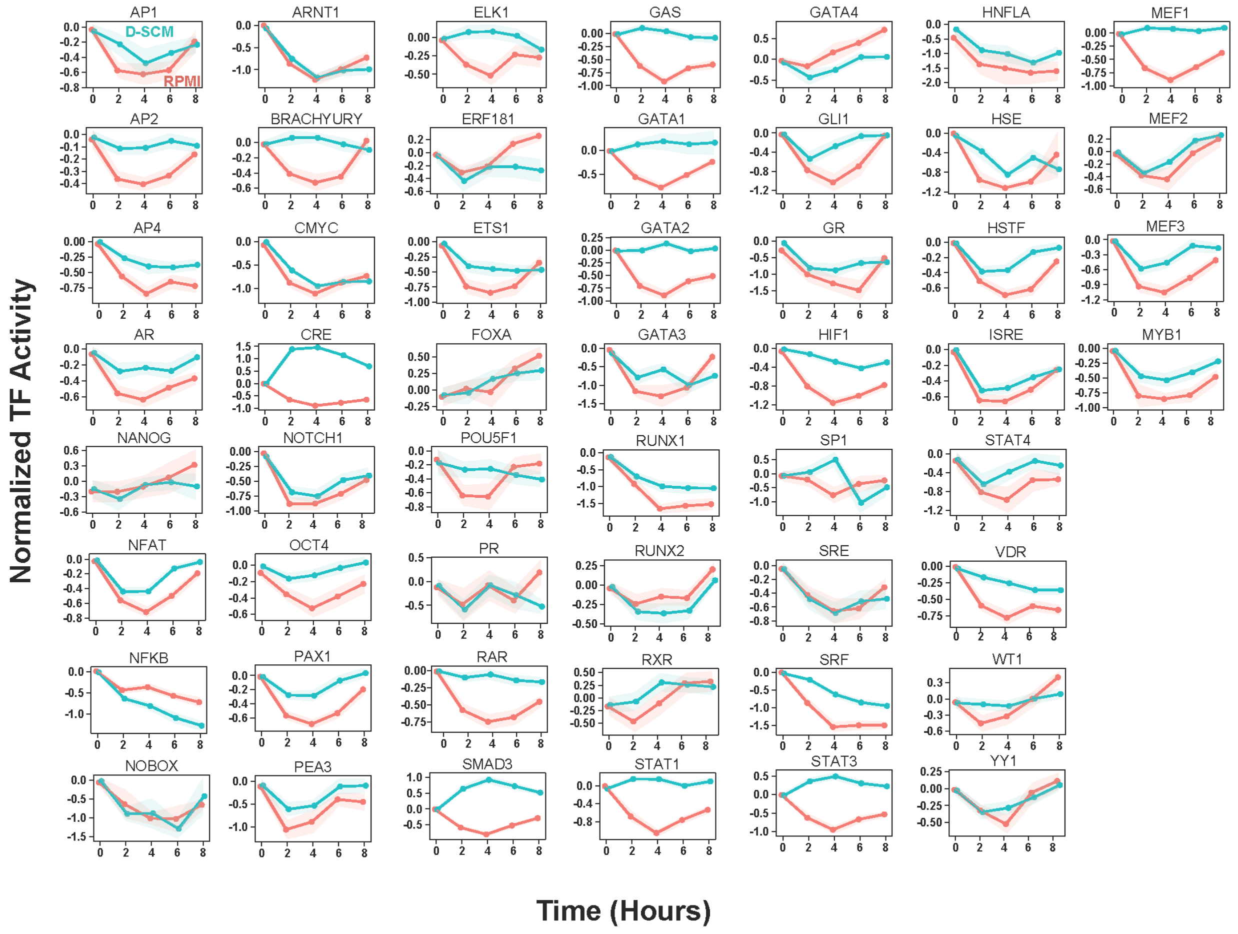

Supplementary Figure 2 – TF activity of MDA-MB-231 cells measured cultured in D-SCM using TRACER. TRACER line-plot data showing 52 TF reporter activity profiles for MDA-MB-231 cells cultured in D-SCM or RPMI over an 8 hour period normalized to control TA-FLuc activity. Red line plots = RPMI, blue line plots = D-SCM (*n* = 6 arrays).

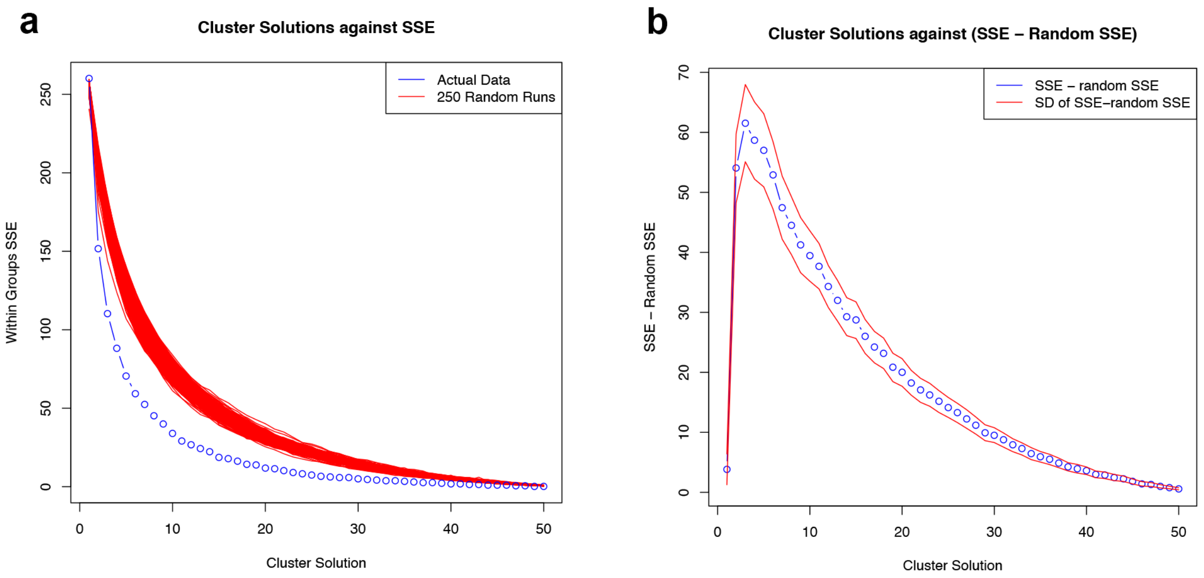

Supplementary Figure 3 - *K*-cluster analysis of MDA-MB-231 TRACER activity in D-SCM. (**a**) Cluster solutions against sum of squared errors compared to 250 random data set runs. (**b**) Absolute difference between actual and random (mean of all runs) SSE against cluster solutions.

Cluster	Reporter	TF Name	Category	Associated GO Processes
1	CRE	cAMP Response Element Binding Protein 1	Canonical Pathways	cAMP response, cell proliferation, migration, invasion
1	STAT3	Signal Transducer and Activator of Transcription 3	Inflammatory Response	Acute phase response, proliferation, cytokine response
1	SMAD3	Smad3	Canonical Pathways	TGFβ pathway response, cell cycle, cell invasion
1	STAT1	Signal Transducer and Activator of Transcription 1	Inflammatory Response	Interferon response, proliferation, cytokine response
1	SRF	Serum Response Factor	Canonical Pathways	MAPK pathway, cell cycle, growth, migration, apoptosis
1	MEF1	Myocyte-specific enhancer factor 1	Differentiation/Development	Mesodermal differentiation, cell adhesion
1	HIF1	Hypoxia Inducible Factor 1A	Differentiation/Development	Hypoxia response, angiogenesis
1	GATA2	Gata binding protein 2	Differentiation/Development	Endothelial; adipocyte differentiation, angiogenesis
1	GATA1	Gata binding protein 1	Differentiation/Development	Hematopoietic differentiation
1	GAS	Interferon-Gamma Activated Sequence	Inflammatory Response	STAT family promoter

Supplementary Table 2 - List of significantly active transcription factor reporters in Cluster 1 of D-SCM TRACER screen. TFs are listed by TF reporter category and associated gene ontology (GO) processes. Sources: TRANSFAC, MetaCore, NCBI databases.

Secreted Factor	Downstream Receptor(s)	Downstream TFs
Calgranulin A (S100a8)	TLR4<br>RAGE	cJun<br>GATA1<br>GATA4<br>HIF1A<br>HSF1<br>IRF3<br>RUNX1<br>RXR<br>SMAD3<br>VDR
Calgranulin B (S100a9)	RAGE	cJun<br>GATA1<br>GATA4<br>HIF1A<br>HSF1<br>RUNX1<br>RXR<br>SMAD3<br>VDR
Haptoglobin	CCR2	AR<br>HIF1A<br>IRF3<br>MafB<br>p73<br>RAR<br>SMAD3<br>SRF<br>STAT1<br>STAT3<br>VDR
Heme Binding Protein	FPR<br>FPRL1	cFos<br>cMyc<br>ENO1<br>SMAD3<br>STAT1<br>SRF<br>STAT3<br>STAT5
Myeloperoxidase	$\alpha_M\beta_2$	AR<br>HIF1A<br>IRF3<br>MafB<br>p73<br>RAR<br>SMAD3<br>VDR

Supplementary Table 3 - List of curated interactions. Interactions between candidate secreted factors, downstream receptors, and downstream TFs identified using MetaCore are catalogued.

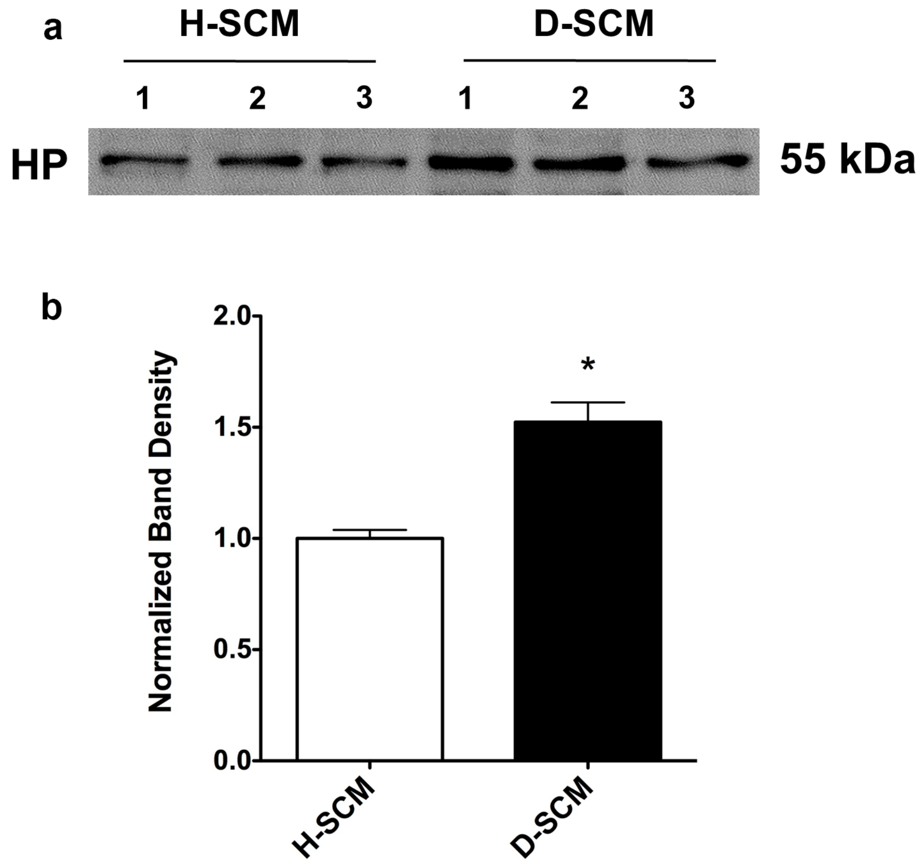

Supplementary Figure 4 – Validation of haptoglobin abundance in H-SCM and D-SCM. (**a**) Western blot of H-SCM and D-SCM samples in triplicate showing Hp bands. (**b**) Corresponding normalized band density quantification ($n = 3$, * $p < 0.006$ compared to H-SCM).

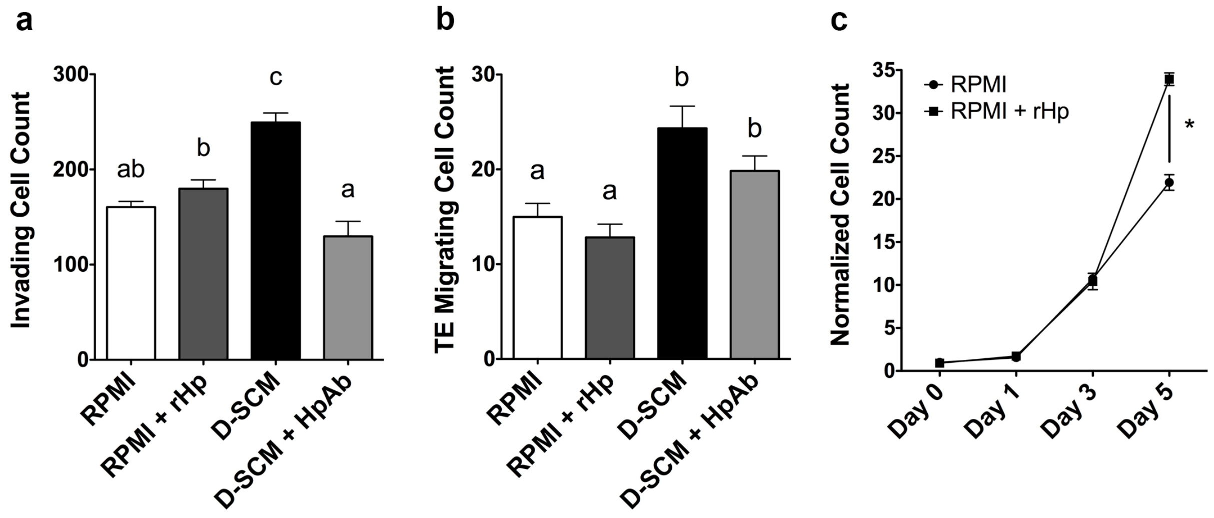

Supplementary Figure 5 - Effects of haptoglobin mediating MDA-MB-231 phenotypes *in vitro*. Tumor cell (**a**) invasion and (**b**) TE migration of MDA-MB-231 cells cultured in control RPMI, RPMI supplemented with rHp, D-SCM, and D-SCM supplemented with HpAb ($n = 8$). Letters above each data column represent statistical significance, with different letters signifying distinct statistical groups ($p < 0.05$). (**c**) MDA-MB-231 cell proliferation measured using an MTS assay ($n = 4$, $*p < 0.05$ compared to RPMI).

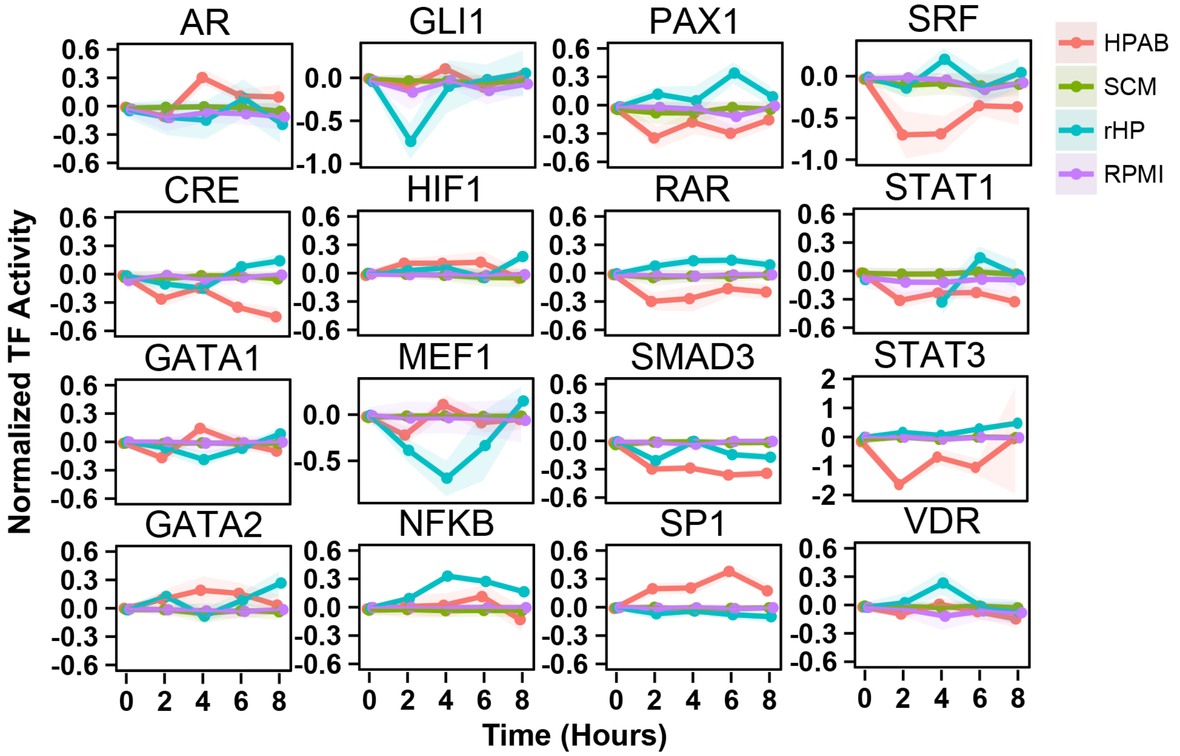

Supplementary Figure 6 - TF activity of MDA-MB-231 cells cultured in various media measured using TRACER. TRACER line-plot data showing 16 TF reporter activity profiles for MDA-MB-231 cells cultured in RPMI, RPMI + rHp, D-SCM, and D-SCM + HpAb media over an 8 hour period, normalized to control TA-FLuc activity (*n* = 6 arrays).

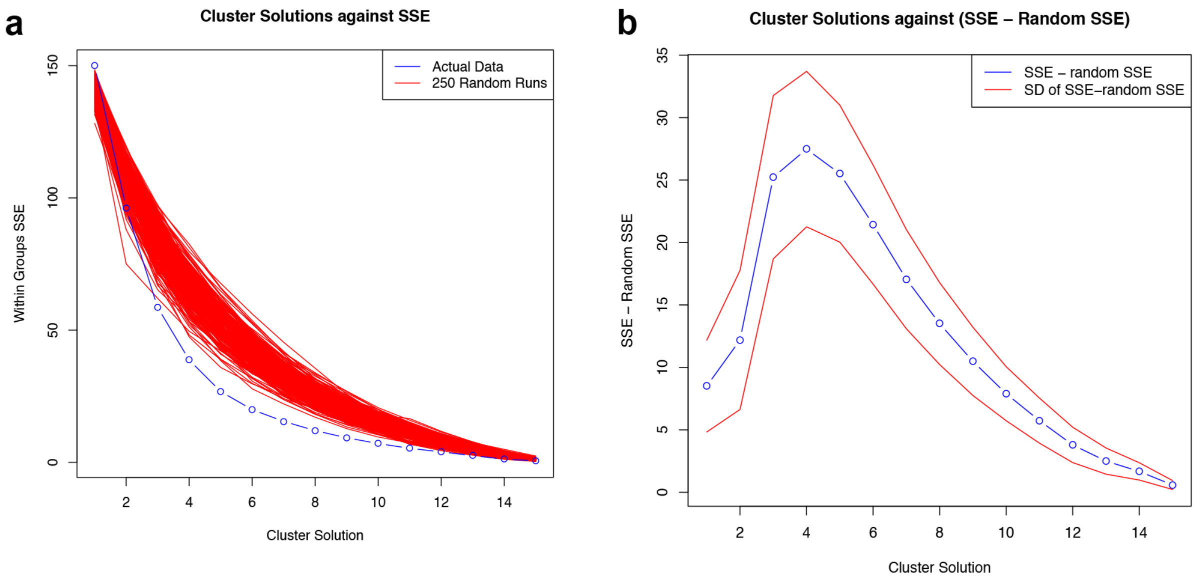

Supplementary Figure 7 - *K*-clustering analysis of MDA-MB-231 TRACER activity cultured in RPMI vs. rHp and D-SCM vs. D-SCM + HpAb media. (**a**) Cluster solutions against sum of squared errors compared to 250 random data set runs. (**b**) Absolute difference between actual and random (mean of all runs) SSE against cluster solutions.
